# Basal cisternostomy as an adjunct to decompressive hemicraniectomy in moderate to severe traumatic brain injury: a systematic review and meta-analysis

**DOI:** 10.1007/s10143-024-02954-4

**Published:** 2024-10-02

**Authors:** Olga Ciobanu-Caraus, Veronica Percuoco, Anna-Sophie Hofer, Martina Sebök, Menno R. Germans, Markus F. Oertel, Luca Regli, Carlo Serra, Victor E. Staartjes

**Affiliations:** 1 Machine Intelligence in Clinical Neuroscience & Microsurgical Neuroanatomy (MICN) Laboratory, Department of Neurosurgery, Clinical Neuroscience Center, University Hospital Zurich, University of Zurich, Rämistrasse 100, Zürich, 8091 Switzerland; 2 Department of Neurosurgery, Medical University Innsbruck, Innsbruck, Austria

**Keywords:** Traumatic brain injury, Head injury, Basal cisternostomy, Cisternal drainage, Decompressive hemicraniectomy, Intracranial pressure

## Abstract

**Background:**

Basal cisternostomy (BC) is a surgical technique to reduce intracranial hypertension following moderate to severe traumatic brain injury (TBI). As the efficacy and safety of BC in patients with TBI has not been well-studied, we aim to summarize the published evidence on the effect of BC as an adjunct to decompressive hemicraniectomy (DHC) on clinical outcome following moderate to severe TBI.

**Methods:**

A systematic literature review was carried out in PubMed/MEDLINE and EMBASE to identify studies evaluating BC as an adjunct to decompressive hemicraniectomy (DHC) in moderate to severe TBI. Random effects meta-analysis was performed to calculate summary effect estimates.

**Results:**

Eight studies reporting on 1345 patients were included in the qualitative analysis, of which five (1206 patients) were considered for meta-analysis. Overall, study quality was low and clinical heterogeneity was high. Adjuvant BC (BC + DHC) compared to standalone DHC was associated with a reduction in the length of stay in the ICU (Mean difference [MD]: -3.25 days, 95% CI: -5.41 to -1.09 days, *p* = 0.003), significantly lower mean brain outward herniation (MD: -0.68 cm, 95% CI: -0.90 to -0.46 cm, *p* < 0.001), reduced odds of requiring osmotherapy (OR: 0.09, 95% CI: 0.02 to 0.41, *p* = 0.002) as well as decreased odds of mortality at discharge (OR 0.68, 95% CI: 0.4 to 0.96, *p* = 0.03). Adjuvant BC compared to DHC did not result in higher odds of a favourable neurological outcome (OR = 2.50, 95% CI: 0.95–6.55, *p* = 0.06) and did not affect mortality at final follow-up (OR: 0.80, 95% CI: 0.17 to 3.74, *p* = 0.77).

**Conclusion:**

There is insufficient data to demonstrate a potential beneficial effect of adjuvant BC. Despite some evidence for reduced mortality and length of stay, there is no effect on neurological outcome. However, these results need to be interpreted with caution as they carry a high risk of bias due to overall scarcity of published clinical data, technical variations, methodological differences, limited cohort sizes, and a considerable heterogeneity in study design and reported outcomes.

**Supplementary Information:**

The online version contains supplementary material available at 10.1007/s10143-024-02954-4.

## Introduction

With over 50 million people affected annually, moderate to severe traumatic brain injury (TBI) is a substantial cause of morbidity and mortality on a global scale [1]. Approximately 60% of patients with moderate or severe TBI suffer severe long-term disability or death [2, 3]. The mechanisms of TBI are mediated by two principal components: primary insult related to the impact of trauma itself, which consists of structural damage such as axonal shearing injury or intracranial hemorrhage, and secondary brain injuries, which are a result of a complex interplay of metabolic, cytotoxic and vasogenic factors that lead to an increase in the intracranial pressure (ICP) and are responsible for excess neurological morbidity following TBI [4].

Since its introduction by Theodor Kocher in the early 20th century, decompressive hemicraniectomy (DHC) has been the standard surgical procedure for the treatment of refractory post-traumatic intracranial hypertension (in patients with diffuse injury and swelling with or without mass lesion, with midline shift or impending herniation, usually Marshall ≥ 3) [5, 6]. Based on the Monroe-Kellie doctrine, the underlying rationale of DHC consists in expanding the volume of the cranial vault to combat rising intracranial pressure (ICP) [7].

The RESCUEicp randomized controlled trials showed that DHC resulted in a reduction of the overall mortality rate but also in higher rates of vegetative state and neurological disability at 6 months follow-up [8]. Various other treatment options have been investigated but did not show a clinical benefit when evaluated in RCTs [9–14]. With a track record of over 200 failed clinical trials, new treatment options for severe TBI are desperately needed [15, 16].

Recent laboratory studies have provided novel mechanistic insights into CSF pathophysiology [17–20]. One model assumes that CSF is absorbed via the pericapillary Virchow-Robin spaces (VRS) at the basal cisterns, which are part of the glymphatic system [17–22]. Increased cisternal pressure following the obstruction of the VRS – such as due to traumatic subarachnoid haemorrhage (tSAH) – may lead to a reversal of CSF flow from the subarachnoid space back into the brain parenchyma (known as the “CSF-shift edema” hypothesis), resulting in increased ICP, disruption of the permeability of the glymphatic system and stagnation of toxic metabolic factors [23–25].

This emerging concept provided the rationale for Cherian et al. to introduce basal cisternostomy (BC) with basal cisternal drainage in 2013 as a novel treatment modality in moderate to severe traumatic brain injury [26]. Following the promising results of their study, this technique has been increasingly adopted by several neurosurgeons worldwide [27–31]. The procedure usually consists of a craniotomy and fenestration of all basal cisterns including the prepontine cistern, passive CSF drainage, and leaving in place an external cisternal drain within the prepontine cistern [32]. However, with few published studies and without any systematic evidence summary, the effect of BC on clinical outcome following TBI remains unclear [30, 33–38]. The present review aims to evaluate the effect of BC as an adjunct to decompressive hemicraniectomy (termed “adjuvant BC” = BC + DHC) in patients with moderate to severe traumatic brain injury (TBI).

## Materials and methods

### Overview

A systematic review was carried out to identify any studies in patients with moderate to severe TBI and reporting intra-/perioperative, or clinical outcomes of adjuvant BC (BC + DHC), including (1) mean Glasgow Outcome Scale (GOS) at follow-up, (2) GOS ≥ 5 at follow-up, (3) mortality at follow-up, (4) complications, (5) operative time, (6) mean ICP (preoperative / after the first burr hole / after hemicraniectomy and/or cisternostomy / at closure / in the ICU), (7) mean brain outward herniation, (8) osmotherapy, (9) complications, and (10) length of stay in the ICU. Title and abstract screening, full-text review, and data extraction were handled independently by three reviewers (VES, VP and OC) using Covidence (Covidence systematic review software, Melbourne, Australia) [39], and disagreements at any stage were resolved by discussion and consensus. This systematic review followed the methodological framework described by Arksey and O’ Malley for Systematic Reviews [40] and the PRISMA Statement [41].

### Search strategy

The PubMed/MEDLINE and EMBASE database was searched to identify eligible articles. The search strategy was as follows for both databases: TBI OR “traumatic brain injury” OR “head injury” OR “brain trauma”) AND (cisternostomy OR cisternal OR cisterns). Word variations and exploded medical subject headings were searched for whenever feasible. References were screened to identify additional relevant articles. The search included articles published between 1995 and 2023 as we aimed to include patients managed under contemporary TBI guidelines and the technique was first described in 2007. Last comprehensive search was conducted on December 26th, 2023.

### Study selection

Only in vivo studies in English enrolling humans aged ≥ 18 years were considered. Systematic reviews, case reports, small case series with less than 5 patients and studies dealing reporting data exclusively on treatment of mild TBI as classified by GCS were excluded. Studies looking at patients with moderate to severe TBI receiving adjuvant BC (BC + DHC) were included. To be eligible for inclusion, studies had to assess at least one of the abovementioned variables of interest at preoperative, intraoperative and postoperative timepoints. The primary comparison of interest was adjuvant BC (BC + DHC) versus standalone DHC. However, we also included studies looking at (1) adjuvant BC (BC + DHC) as a standalone treatment or (2) comparing adjuvant BC (BC + DHC) versus standalone BC.

### Data extraction and quality assessment

The following data were extracted from all included publications: Study design and year of publication, number of patients, mean patient age and sex distribution, adjuvant BC (BC + DHC) and standalone DHC, technical nuances, as well as at least one of the outcomes of interest. The methodological quality of the included studies was graded using the Newcastle-Ottawa Quality Assessment Scale for Cohort Studies [42] and the Cochrane Risk-of-Bias (RoB-2) tool for RCTs [43].

### Statistical meta-analysis

Based on the anticipated heterogeneity and the lack of larger RCTs for certain outcomes, a random-effects analysis model was applied: Mantel-Haenszel models evaluating odds ratios as effect measure for dichotomous outcomes, and inverse variance models evaluating mean differences as effect measure for continuous outcomes [44]. Cochran’s Q and I^2^ were used to evaluate the strength of evidence for heterogeneity and the level of heterogeneity, respectively. A *p* ≤ 0.1 was considered as relevant heterogeneity. We performed subgroup analyses for RCTs. Reporting bias was assessed by visual inspection of funnel plots. The meta-analyses were carried out in Review Manager 5.4 (The Nordic Cochrane Centre, The Cochrane Collaboration, Copenhagen, Denmark) [45]. *P* ≤ 0.05 on 2-tailed tests were considered statistically significant for the assessment of overall effect.

## Results

### Literature search

**Fig. 1:**
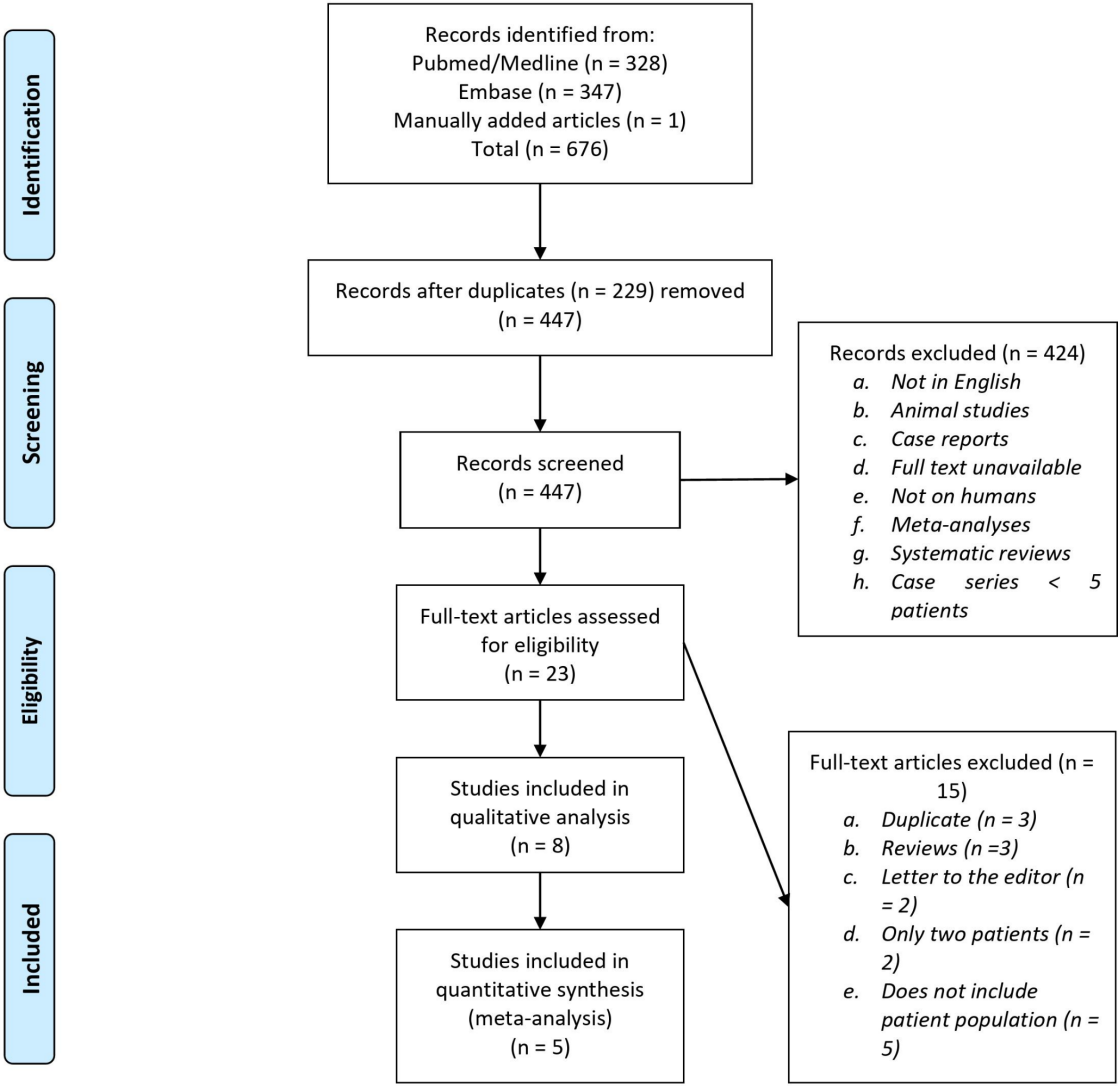
PRISMA Flowchart

A PRISMA flowchart is shown in Fig. 1. Of ten publications [27–29, 46–52] which originally met the inclusion criteria, Vemula et al. [51] was based on the same cohort as the publication of Chandra et al. [29], and only the latter publication was regarded. This was also excluded since it deals with standalone BC and not to adjuvant BC (BC + DHC) and did not meet inclusion criteria. A total of 8 publications was included for qualitative analysis [27, 28, 46–50, 52]. A total of five articles had sufficient outcome data in appropriate format to be eligible for quantitative meta-analysis [27, 46, 48, 50, 52].

### Included study characteristics and quality

Tables 1 and 2 summarizes the characteristics of the included studies and their quality assessment while clinical outcomes are detailed in Tables 3 and 4. Details of the included study characteristics are provided in Supplementary Content 1 – Supplementary Table 1.

**Table 1:** Overview of the study-related characteristics of the included studies

Author	No. Pts.	Intervention	Study type	Inclusion criteria	Exclusion criteria	Indication	Pathology	Newcastle-Ottawa Scale (S/C/O)	Cochrance Risk of Bias Tool
**Adjuvant BC (BC + DHC) vs. standalone DHC**									
**Kumar et al. 2022** [48]	40	*BC + DHC*: 9 *DHC*: 31	Prospective; Quasi-experimental (according to which surgeon was on call); Single-center	Thickness ≥ 10 mm Midline shift ≥ 5 mm	-	*BC + DHC*: Mild TBI: 2 (22.2%) Moderate TBI: 5 (55.6%) Severe TBI: 2 (22.2%) *DHC*: Mild TBI: 5 (26.2%) Moderate TBI: 10 (32.2%) Severe TBI *n* (%): 16 (51.6%)	*BC + DHC* aSDH: 2 (22%) Contusions: 5 (55.6%) aSDH + Contusions: 2 (22.2%) Midline shift in mm: 8.6 ± 1.5 *DHC*: aSDH: 6 (19.4%) Contusions: 15 (48.4%) aSDH + Contusions: 10 (32.2%) Midline shift in mm: 7.6 ± 2.4	3/1/2	NA
**Youssef et al.**, **2020** [52]	40	*BC + DHC*: 20 *DHC*: 20	Prospective; RCT; single-center	aSDH Thickness ≥ 10 mm Midline shift ≥ 5 mm GCS < 10	-	-	-	NA	High
**Singh et al.**, **2021** [50]	54	*BC + DHC*: 27 *DHC*: 27	Prospective; RCT; single-center	*Total*: aSDH Severe TBI (GCS ≤ 7)	-	-	-	NA	High
**Giammattei et al.**, **2020** [27]	40	*BC + DHC*: 18 *DHC*: 22	Retrospective; single-center	*BC + DHC*: Severe TBI (GCS ≤ 8) aSDH, aEDH, aICH ≥ 10 mm *DHC*: Severe TBI (GCS ≤ 8) aSDH ≥ 10 mm	Brainstem dysfunction/signs of irreversible brain damage Severe hemodynamic instability Hemorrhagic diathesis	*BC + DHC*: Midline shift (mm ± SD): 10.8 ± 6.4 *DHC*: Midline shift (mm ± SD): 7.1 ± 6.3	-	4/1/3	NA
**Adjuvant BC (BC + DHC) vs. standalone BC**									
**Parthiban et al.**, **2021** [28]	40	*BC + DHC*: 13 *BC*: 27	Retrospective; single-center, single surgeon	*-*	*-*	Mild TBI: 3 (7.5%) Moderate TBI: 8 (20%) Severe TBI: 29 (72.5%)	-	4/0/2	NA
**Encarnación Ramirez et al.**, **2023** [49]	30	*BC + DHC: 24* *BC: 6*	Prospective; multicenter	Age 18–70 years Severe TBI (GCS ≤ 8) DSH > 20 mm or Midline shift > 5 mm on CT	GCS 3 Moderate and mild TBI Axonal diffuse injury Cardiopulmonary instability Infarcts of brainstem and multi-organ injuries	-	-	4/0/3	NA
**Adjuvant BC (BC + DHC) vs. standalone BC vs. standalone DHC**									
**Cherian et al.**, **2019** [46]	1032	*BC + DHC*: 272 *BC*: 476 *DHC*: 284	Retrospective; single-center	*BC*: MPC score 3–7 (scale 0–10): GCS motor score M4 Anisocoria (unilaterally dilatative and reactive pupil) Obliteration of suprasellar cisterns/cerebellopontine angle cistern widening Oculomotor nerve palsy tSAH with brain swelling aSDH, EDH with brain swelling or mass effect	> 80 years of age Hemorrhagic diathesis (International Normalized Ratio > 3)	*-*	-	3/0/2	NA
**Adjuvant BC (BC + DHC) standalone**									
**Goyal and Kumar**, **2021** [47]	9	*BC* + *DHC*: 9	-	-	-	Moderate TBI: 3 (33%) Severe TBI: 5 (55%)	aSDH ≥ 10 mm: 2 (22%) multiple contusions with mass effect: 5 (55%) aSDH + multiple contusions: 2 (22%) Midline shift ≥ 5 mm: 9 (100%) Mean midline shift in mm: 8.61	4/0/2	NA

aSDH acute subdural hematoma; BC, basal cisternostomy; DHC, standalone decompressive hemicraniectomy; BC + DHC, adjuvant basal cisternostomy + decompressive hemicraniectomy; EDH, epidural hematoma; ICH, intracerebral hematoma, RCT, randomized controlled trial; SD, standard deviation; TBI traumatic brain injury; tSAH, traumatic subarachnoid hemorrhage

**Table 2:** Overview of the baseline clinical and surgical characteristics of the included studies

Author	Age, mean (± SD/range)	Male, *n* (%)	Marshall score, mean (± SD)	Rotterdam score, mean (± SD)	Anisocoria, *n* (%) FND, *n* (%)	Duration of surgery (mins.)	Technical nuances
**Adjuvant BC (BC + DHC) vs. standalone DHC**							
**Kumar et al.**, **2022** [48]	*BC + DHC*: 45.7 ± 14.0 *DHC*: 39.2 ± 16.0	*BC + DHC*: 6 (67%) *DHC*: 21 (67%)		*BC + DHC*:3.4 ± 0.5 *DHC*:3.2 ± 0.7	*BC + DHC*: Anisocoria: 2 (22.2%) Hemiparesis: 3 (33.3%) *DHC*: Anisocoria. 10 (32.2%) Hemiparesis: 12 (38.7%)		*Cisterns opened*: Partial cisternostomy: Interoptic, Opticocarotid, Lateral carotid Complete cisternostomy- Interoptic, Opticocarotid, Lateral carotid, Prepontine *Drainage*: ICP catheter and cisternal drain was kept in place for 72 h
**Youssef et al.**, **2020** [52]	*BC + DHC*: 39.7 ± 15.5 *DHC*: 41.2 ± 14.3	*BC + DHC*: 17 (85%) *DHC*: 18 (90%)		-		*BC + DHC*: 175.5 ± 17 *DHC*: 140 ± 11.7	*Cisterns opened*: Interoptic, Opticocarotic, Lateral carotid *Liliequist membrane*: Incised through opticocarotid or lateral carotid window
**Singh et al.**, **2021** [50]	*BC + DHC*: *50.33 ± 17* *DHC*: *42.25 ± 11*	*BC + DHC*: 103 ± 43 *DHC*:72 ± 32		-	*BC + DHC*: Anisocoria: 21 (77%) *DHC*: Anisocoria: 21 (77%)		-
**Giammattei et al.**, **2020** [27]	*BC + DHC*: 49.9 ± 19 *DHC*: 48.4 ± 20.4	*BC + DHC*: 12 (66.7%) *DHC*:18 (81.8%)		*BC + DHC*:4.7 *DHC*:3.8	*BC + DHC*: Anisocoria: 10 (55%) *DHC*: Anisocoria: 8 (36%)	*BC + DHC*: 204 ± 43 *DHC*: 178 ± 30.5	*Cisterns opened*: Opticocarotid *Liliquist membrane*: Incised through opticocarotid cistern along with the lamina terminalis *Drainage*: CSF drainage was kept in place for a mean of 7.2 ± 3.3 days; Drainage of 207 mL/day
**Adjuvant BC (BC + DHC) vs. standalone BC**							
**Parthiban et al.**, **2021** [28]	44.3 (range 17–70)	31 (77.5%)		-			*Cisterns opened*: Opticocarotid, Lateral carotid *Liliquist membrane*: Incised up to prepontine and infratentorial cisterns *Drainage*: Cisternal drainage was kept in place for 5–7 days; Drainage of 100–150 ml/day
**Encarnación Ramirez et al.**, **2023** [49]	-	9 (30%)		-			*Cisterns opened*: Sylvian, Chiasmatic, Opticocarotid, Lateral carotid *Liliquist membrane* Incised through the opticocarotid or lateral carotid window *Drainage*: Cisternal drainage was kept in place for 3–5 days; Drainage of 150–200 ml/day
**Adjuvant BC (BC + DHC) vs. standalone BC vs. standalone DHC**							
**Cherian et al.**, **2019** [46]	-	-	-	-	-	-	-
---**Adjuvant BC (BC + DHC) standalone**							
**Goyal and Kumar**, **2021** [47]	45.7 ± 14.04 (range 25–72)	5 (55.6%)	-	3.4 ± 0.53 (range 3–4)	Anisocoria: 1 (11%) Contralateral hemiparesis: 3 (33.33%)	*Additional time for cisternostomy*: *Complete cisternostomy*: 20.0 ± 15.0 *Partial cisternostomy*: 34.0 ± 16.2	*Cisterns opened*: *Complete cisternostomy*: 5 Interoptic, opticocarotid, lateral carotid, prepontine *Partial cisternostomy*: 5* interoptic, opticocarotid, lateral carotid *Drainage*: Cisternal drain was removed after 72 h.

BC, basal cisternostomy; DHC, standalone decompressive hemicraniectomy; BC + DHC, basal cisternostomy + decompressive hemicraniectomy; CSF, cerebrospinal fluid; FND, focal neurological deficits; ICP, intracranial pressure, SD, standard deviation

**Table 3:** Overview of outcomes related to neurological outcome, mortality, complications, brain outward herniation and patients requiring osmotherapy among the included studies

Author	GCS at admission, mean (± SD)	GCS at discharge, mean (± SD)	GOS at follow-up, mean (± SD) or (%)	Mortality at discharge, *n* (%)	Mortality at follow-up, *n* (%)	Complications	Brain outward herniation, mean in cm (± SD)	Patients requiring osmotherapy, *n* (%)
**Adjuvant BC (BC + DHC) vs. standalone DHC**								
**Kumar et al.**, **2022** [48]	*BC + DHC*: 7.9 ± 3.1 *DHC*: 8.0 ± 3.4	*BC + DHC*: 11.7 ± 2.9 *DHC*: 10.5 ± 3.7	*At 30 days* *GOS-E ≥ 5* (range 1–8) *BC + DHC*: 1 (11.1%) *DHC*: 3 (9.7%)	-	*At 30 days* *BC + DHC*: 6 (66.6%) *DHC*: 10 (32.2%)	*BC + DHC*: ICA injury: 1 (11.1%) contralateral cisternostomy following refractory intracranial hypertension: 1 (11.1%) *DHC*: 0	-	-
**Youssef et al.**, **2020** [52]	*BC + DHC*:6.1 ± 2 *DHC*: 6.4 ± 1.6	-	*At 4 weeks* *BC + DHC*: 2.95 ± 1.67 *DHC*: 2.40 ± 1.73	-	*At 4 weeks* *BC + DHC*: 7 (35%) *DHC*: 10 (50%)	-	-	-
**Singh et al.**, **2021** [50]	*BC + DHC*:5.56 ± 1.22 (range 3–7) *DHC*:5.52 ± 1.28 (range 3–7)	-	*At 6 months* *GOS ≥ 5* (range 1–5) *BC + DHC*: 4 (14.81%) *DHC*: 2 (7.4%)	*BC + DHC*: 2 (7.4%) *DHC*: 3 (11.1%)	*At 6 months* *BC + DHC*: 3 (11.1%) *DHC*: 9 (33.3%)	-	*BC + DHC*: 0.47 ± 0.56 *DHC*: 1.2 ± 0.32	*BC + DHC*: 3 (11%) *DHC*:20 (74%)
**Giammattei et al.**, **2020** [27]	*BC + DHC*: 5.8 *DHC*: 5.7	*BC + DHC*: 13.1 *DHC*: 10.4	*At 6 months* *Mean* *BC + DHC*: 4.8 ± 2.5 *DHC*: 3.6 ± 2.1 *GOS-E ≥ 5* (range 1–8) *BC + DHC*: 11 (61%) *DHC*: 7 (35%)	*BC + DHC*: 4 (22%) *DHC*: 6 (27%)	-	*BC + DHC*: subcutaneous hematomas with evacuation: 2 (7.4%) *DHC*: enlargement of mass lesion with surgical evacuation: 1 (4.5%) contusion blossoming without mass effect: 1 (4.5%) high ICP and EVD placement: (4.5%)	*BC + DHC*: 0.37 ± 0.85 *DHC*: 0.86 ± 0.67	*BC + DHC*: 3 (16.7%) *DHC*: 11 (50%)
**Adjuvant BC (BC + DHC) vs. standalone BC**								
**Parthiban et al.**, **2021** [28]	*Total*: 7	*Total*: 13	*At 6 months* *GOS ≥ 4* (range 1–5) *BC*: 23 (85%) *BC + DHC*: 10 (76.9%)	-	-	*Total*: wound infections: 2 (5%) hydrocephalus: 5 (12.5%)	-	-
**Encarnación Ramirez et al.**, **2023** [49]	*Total*: 5.9 (range 4–8)	-	*At 6 months* *GOS-E ≥ 5* *BC*: 5 (100%) *BC + DHC*: 19 (91.7%)	*BC + DHC: 3* (12.5%) *BC: 1* (16.7%)	-	-	-	-
**Adjuvant BC (BC + DHC) vs. standalone BC vs. standalone DHC**								
**Cherian et al.**, **2019** [46]	-	-	*At 6 weeks* *BC*: 3.9 *BC + DHC*: 3.7 *DHC*: 2.8	*BC + DHC*: 72 (26.4%) *BC*: 74 (15.6%) *DHC*: 99 (34.8%)	-	-	-	-
**Adjuvant BC (BC + DHC) standalone**								
**Goyal and Kumar**, **2021**[47]	7.89 ± 3.06 median: 8 (range 4–14)	At 72 h*: 5.67 ± 3.78	-	-	-	ICA injury: 1 (11.1%) refractory postoperative ICP and contralateral cisternostomy: 1 (11.1%)	-	-

BC, basal cisternostomy; DHC, standalone decompressive hemicraniectomy; BC + DHC, adjuvant basal cisternostomy + decompressive hemicraniectomy; EVD, external ventricular drainage; GCS, Glasgow Coma Scale; GOS, Glasgow Outcome Scale; GOS-E, Glasgow Outcome Scale Extended; ICA, internal carotid artery; ICP, intracranial pressure; SD, standard deviation;

* Not reported in patients 2, 4 and 7. Patient 2 experienced a cardiac arrest at 32 h when cisternal and parenchymal pressures dropped to 27 mmHg and 14 mmHg respectively. Patient 4 experienced a cardiac arrest at 56 h when cisternal and parenchymal pressures fell to 22 mmHg and 13 mmHg respectively. Patient 7 experienced a cardiac arrest after the second surgery, when cisternal and parenchymal pressure fell to 58 mmHg and 72 mmHg respectively

**Table 4:** Overview of outcomes related to intracranial pressure, length of stay, and cranioplasty among the included studies

Author	ICP preoperative or after the first burr hole in mmHg, mean (± SD)	ICP after durotomy in mmHg, mean (± SD)	ICP after hemicraniectomy and/or cisternostomy in mmHg, mean (± SD)	Closing ICP in mmHg, mean (± SD)	ICP in the ICU in mmHg, mean (± SD)	ICU stay in days, mean (± SD)	Hospital stay in days, mean (± SD)	Time to cranioplasty in days, mean (± SD)
**Adjuvant BC (BC + DHC) vs. standalone DHC**								
**Kumar et al.**, **2022** [48]	-	*BC + DHC*: 25.7 ± 10.5 *DHC*:25.4 ± 12.2	-	*BC + DHC*: 25.7 ± 10.5 *DHC*: 25.4 ± 12.2	*First 72 h*: *BC + DHC*: 11.9 ± 2.1 *DHC*: 11.7 ± 1.5	*BC + DHC*: 7.0 ± 6.1 *DHC*: 10.5 ± 9.3	*BC + DHC*: 15.0 ± 20.2 *DHC*: 19.3 ± 13.9	-
**Youssef et al.**, **2020** [52]	-	-	-	-	-	*BC + DHC*: 7.8 ± 7 *DHC*: 10.3 ± 6	-	-
**Singh et al.**, **2021** [50]	-	-	-	-	-	*BC + DHC*: 12.3 ± 4.2 *DHC*:14.9 ± 9.2	-	-
**Giammattei et al.**, **2020** [27]	*Preoperative in secondary procedure* *BC + DHC*:20.3 ± 1.5 *DHC*:21.3 ± 6.9	-	-	*BC + DHC*: 12 ± 0.8 *DHC*: 16 ± 1.4	*First 24 h*: *BC + DHC*: 11.8 ± 0.7 *DHC*: 16.8 ± 1.8	*BC + DHC*: 11.9 ± 7.3 *DHC*:16.9 ± 7.6	-	*BC + DHC*: 32.5 ± 20.9 *DHC*: 55.6 ± 40.8
**Adjuvant BC (BC + DHC) vs. standalone BC**								
**Parthiban et al.**, **2021** [28]	-	-	-	-	-	-	41 (range 2-180)	-
**Encarnación Ramirez et al.**, **2023** [49]	-	-	-	-	-	4.8	12.2	-
**Adjuvant BC (BC + DHC) vs. standalone BC vs. standalone DHC**								
**Cherian et al.**, **2019** [46]	-	-	-	-	-	*BC + DHC*: 3 *BC*: 2 *DHC*: 6	-	-
**Adjuvant BC (BC + DHC) standalone**								
**Goyal and Kumar**, **2021** [47]	After the first burr hole* 25.70 ± 10.48	-	-	11.30 ± 5.95*	*First 72 h* Cisternal pressure**: 32 ± 19.95 Parenchymal pressure**: 20.17 ± 21.58	-	-	-

BC, basal cisternostomy; DHC, standalone decompressive hemicraniectomy; BC + DHC, adjuvant basal cisternostomy + decompressive hemicraniectomy; ICP, intracranial pressure; ICU, intensive care unit; SD, standard deviation

* Pressures of both the first (left) and second (right) procedure of patient 7 were included in the calculation

**Pressures of both the first (left) and second (right) procedure of patient 7 were included in the calculation. Pressures of patient 1, 3 and 8 were not taken. In patient 1, following ICA injury, it was not considered appropriate to leave a cisternal drain. In patient 3, cisternal pressure could not be monitored due to a technical fault in the monitor. In patient 8 cisternal drain and parenchymal pressure catheter were removed because the patient was very restless

The primary aim of this study was to compare the outcomes of adjuvant BC (BC + DHC) versus standalone DHC. However, for the sake of completion in summarizing the clinical evidence on adjuvant BC in TBI, we also included other secondary comparisons:

- Primary comparison:
  - Adjuvant BC (BC + DHC) versus standalone DHC:
    - Kumar et al., 2022 (40 patients) [48]: Prospective, quasi-experimental study.
    - Giammattei et al., 2020 [27] (40 patients): retrospective single-centre study.
    - Youssef et al., 2020 [52] (40 patients) RCT.
    - Singh et al. 2021 [50] (54 patients): RCT.
- Secondary comparisons:
  - Adjuvant BC (BC + DHC) versus standalone BC:
    - Parthiban et al., 2021 [28] (40 patients): retrospective single-centre study.
    - Encarnación Ramirez et al., [49] (30 patients): prospective, three-centre study.
  - Adjuvant BC (BC + DHC) versus standalone BC versus standalone DHC:
    - Cherian et al., 2019 [46] (1032 patients): Retrospective single-centre study.
  - Adjuvant BC (BC + DHC) standalone:
    - Goyal and Kumar, 2021 [47] (9 patients): Prospective observational study reporting the results o f nine patients treated with adjuvant BC (BC + DHC) standalone.

With regard to quality, most cohort studies analyzed an at least somewhat representative cohort according to the definition of the Newcastle-Ottawa Scale but most suffered from poor comparability and provided too short follow-up data to draw valid conclusions on the final outcome of the patients.

According to the Cochrane risk-of-bias tool, the remaining two RCTs were assessed to be at high risk of bias as the randomization process was not detailed in the study by Singh et al. [50] and was generated by alternation in the study by Youssef et al. [52].

Results of the primary comparison (adjuvant BC = BC + DHC) are reported in the following paragraph. The results of the secondary comparisons are reported separately in Supplementary Content 2 – Supplementary Results 1, which includes the following comparisons: Adjuvant BC (BC + DHC) vs. standalone BC, Adjuvant BC (BC + DHC) versus standalone BC versus standalone DHC and Adjuvant BC (BC + DHC) standalone.

### Primary comparison: adjuvant BC (BC + DHC) versus standalone DHC

#### Intraoperative parameters

##### Operative time

**Fig. 2:**
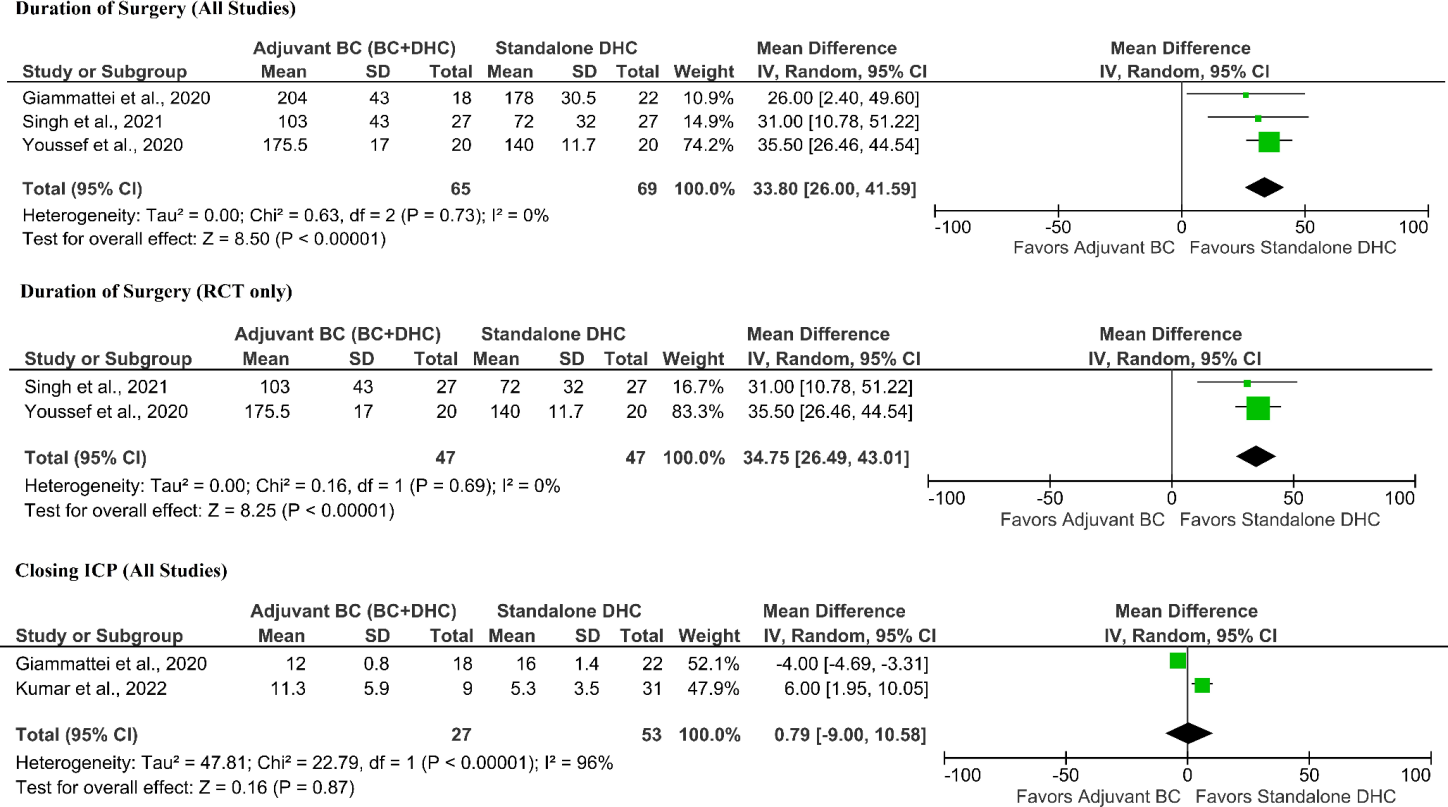
Forest plots detailing the meta-analyses of intraoperative parameters BC + DHC, adjuvant basal cisternostomy + decompressive hemicraniectomy; DHC, standalone decompressive hemicraniectomy; RCT, randomized controlled trial; RCT, randomized controlled trial;

Among two RCTs [50, 52] and a retrospective study [27] with a total of 132 patients reporting duration of surgery (Fig. 2), there was a statistically significant increase in the duration of surgery in patients who underwent adjuvant BC compared to DHC (*Mean difference [MD]*: 33.80 min, *95% confidence interval [95% CI]*: 26.0 to 41.59, *p* < 0.001). Statistical heterogeneity was low with an I^2^ of 0% (*p* = 0.73). Among two RCTs [50, 52] (94 patients), the duration of surgery was longer in patients with adjuvant BC (*MD*: 34.75, *95% CI*: 26.49 to 43.01, *p* < 0.001). Statistical heterogeneity was low with an I^2^ of 0% (*p* = 0.69).

##### Closing ICP

Among a retrospective [27] and prospective study [48] (80 patients) reporting the closing ICP, there was no statistically significant difference in the closing ICP (*MD*: 0.79 mmHg, *95% CI*: -9.00 to 10.58 mmHg, *p* = 0.87). Statistical heterogeneity was considerable with an I^2^ of 96% (*p* < 0.001).

#### Perioperative parameters

##### Length of stay in the ICU

**Fig. 3:**
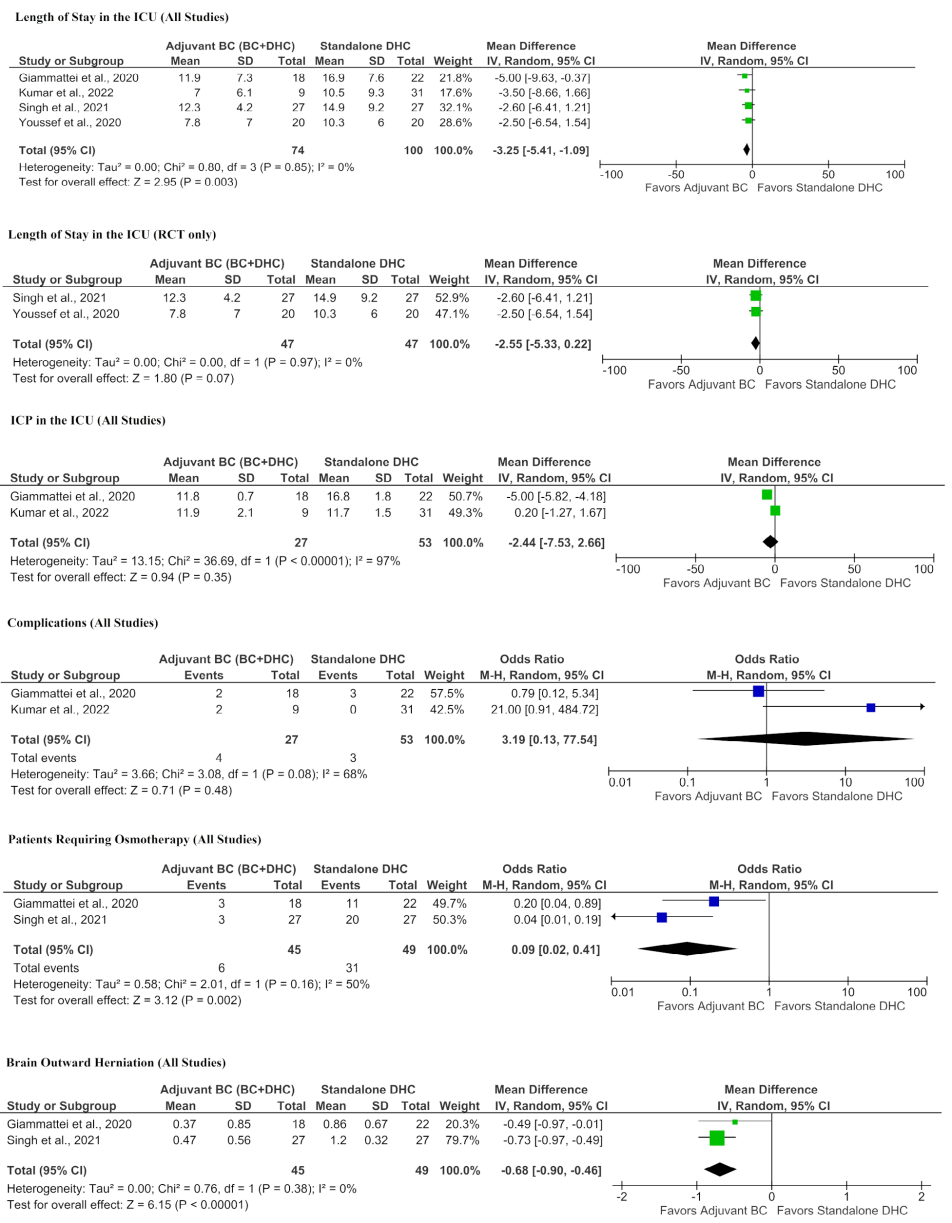
Forest plots detailing the meta-analyses of perioperative parameters BC + DHC, adjuvant basal cisternostomy + decompressive hemicraniectomy; DHC, standalone decompressive hemicraniectomy; RCT, randomized controlled trial; RCT, randomized controlled trial;

There was a statistically significant reduction in the length of ICU stay for adjuvant BC (*MD*: -3.25 days, *95% CI*: -5.41 to -1.09 days, *p =* 0.003, Fig. 3), among two prospective RCTs [50, 52], a prospective [48] and a retrospective [27] study (174 patients) reporting appropriate data. Statistical heterogeneity was low with 0% (*p* = 0.85). Among two RCTs [50, 52] (94 patients), there was no statistically significant intergroup difference (*MD*: 2.55 days, *95% CI*: -5.33 to 0.22 days, *p* = 0.07). Statistical heterogeneity was low with an I^2^ of 0% (*p* = 0.97).

##### ICP in the ICU

Among a prospective and a retrospective study (80 patients) reporting ICP in the ICU, there was no statistically significant intergroup difference (*MD*: -2.44 mmHg, *95% CI*: -7.53 to 2.66 mmHg, *p* = 0.35). Statistical heterogeneity was considerable with an I^2^ of 97% (*p* < 0.001).

##### Complications

Within a prospective [48] and a retrospective study [27] (80 patients) reporting appropriate data, there was no statistical difference in postoperative complications (*OR* 3.19, *95% CI*: 0.13–77.54, *p* = 0.48). Statistical heterogeneity was substantial with an I^2^ of 68% (*p* = 0.08).

##### Osmotherapy

A RCT [50] and a retrospective study [27] reported data on patients requiring osmotherapy (94 patients). Among these, there was a statistically significant reduction in the odds of requiring osmotherapy in patients with adjuvant BC (*Odds ratio [OR]* 0.09, *95% CI*: 0.02 to 0.41, *p* = 0.002). Statistical heterogeneity was moderate with an I^2^ of 50% (*p* = 0.16).

##### Brain outward herniation

Among one RCT [50] and a retrospective study [27] reporting brain outward herniation (94 patients), there was a significant reduction in brain outward herniation (measured as described by Bruno et al. [53]) in patients with adjuvant BC (*MD*: -0.68 cm, *95% CI*: -0.90 to -0.46 cm, *p* < 0.001). Statistical heterogeneity was low with an I^2^ of 0% (*p* = 0.38).

#### Clinical outcomes

##### Mean glasgow outcome scale (GOS) at follow-up

**Fig. 4:**
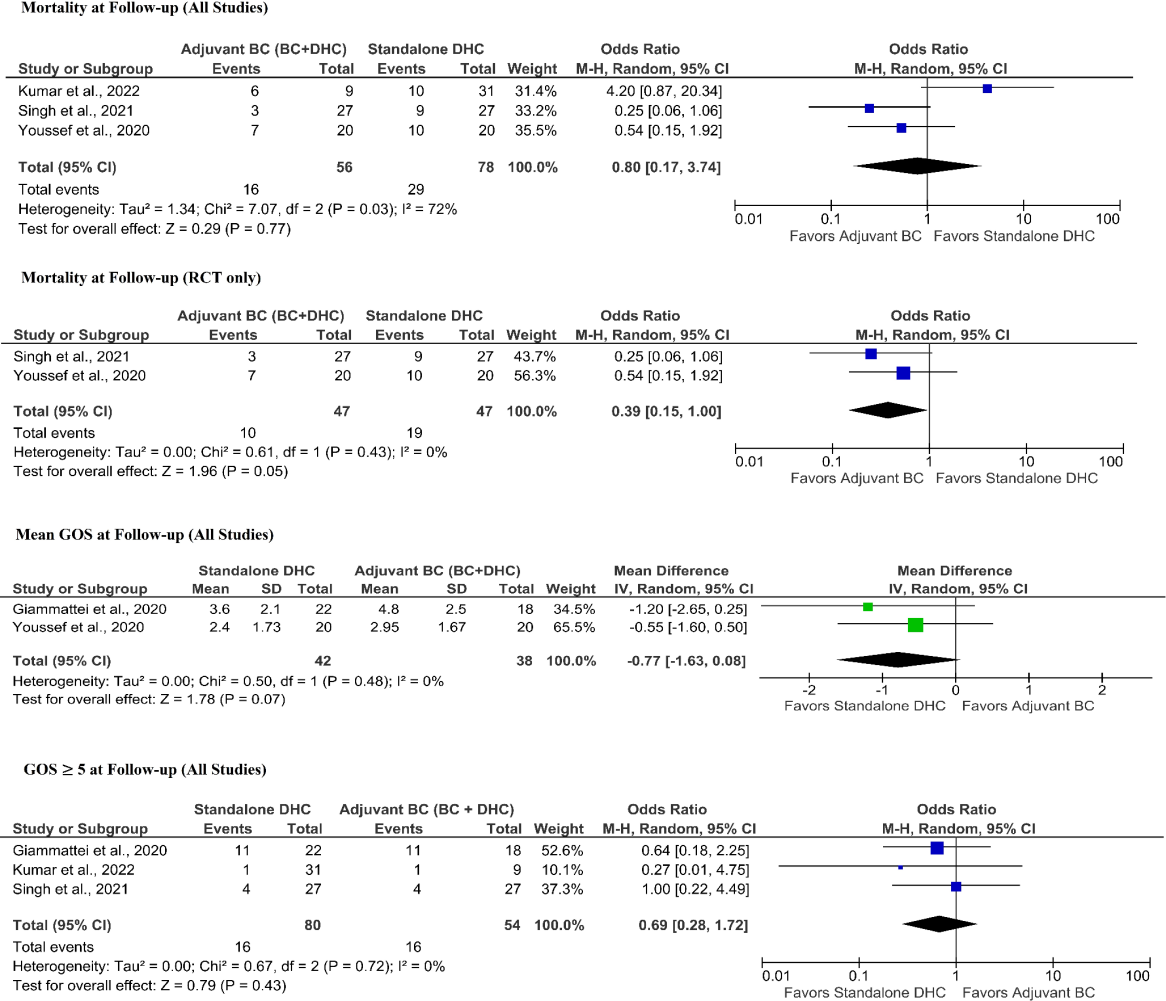
Forest plots detailing the meta-analyses of clinical outcomes BC + DHC, adjuvant basal cisternostomy + decompressive hemicraniectomy; DHC, standalone decompressive hemicraniectomy; RCT, randomized controlled trial; RCT, randomized controlled trial;

Forest plots for meta-analyses of clinical outcomes are summarized in Fig. 4. Among a RCT [52] and a retrospective [27] study (80 patients) reporting mean GOS at final follow-up a median follow-up of four weeks, there was no significant change in patients with adjuvant BC (*MD*: 0.77, *95% CI*: -0.08 to 1.63, *p* = 0.07). Statistical heterogeneity was low with an I^2^ of 0% (*p* = 0.48).

##### GOS ≥ 5 at final follow-up

Among a RCT [50], a prospective [48] and a retrospective [27] study (134 patients) reporting GOS ≥ 5 at a median follow-up of six months, there was no statistically significant intergroup difference (*OR* 2.50, *95% CI*: 0.95 to 6.55, *p* = 0.06).

Statistical heterogeneity was low with an I^2^ of 0% (*p* = 0.74).

##### Mortality at follow-up

Among two RCTs [50, 52] and a prospective study [48] (134 patients), there was no statistical intergroup difference (*OR* 0.80, *95% CI*: 0.17 to 3.74, *p* = 0.77). Statistical heterogeneity was high with an I^2^ of 72% (*p* = 0.03). Among two RCTs [50, 52] (94 patients), patients with adjuvant BC had borderline significantly reduced odds for mortality at a median follow-up of four weeks (*OR* 0.39, *95% CI*: 0.15 to 1.00, *p* = 0.05). Statistical heterogeneity was low with an I^2^ of 0% (*p* = 0.43).

### Publication bias

Funnel plots are provided for each analysis in Supplementary Content 3 – Supplementary Figs. 1–12. Due to the low number of studies, a proper qualitative or quantitative analysis was not possible, and no assumptions can be made regarding publication bias.

## Discussion

The present study is the first systematic review and meta-analysis to quantify the effect of adjuvant BC (BC + DHC) in moderate to severe TBI. We evaluated eight relevant studies including a total of 1305 patients, of which three were RCTs analysing a total of 144 patients. Despite technical variations, methodological differences and limited cohort sizes of the respective studies, the results of our meta-analysis indicate that adjuvant BC (BC + DHC) compared to standalone DHC may potentially be associated with a reduction in the length of stay in the ICU and a lower mean brain outward herniation. In terms of clinical outcome, adjuvant BC (BC + DHC) appeared to reduce in-hospital mortality and long-term mortality at least in randomized studies, while there was no significant effect on neurological outcome.

The principal paradigm in TBI treatment has been to decrease ICP to minimize subsequent secondary brain injury [54–56]. DHC constitutes the standard last-resort surgical procedure for refractory intracranial hypertension that aims at reducing the ICP by providing more space for the brain to expand [57]. Optimization of ICP does not seem to correlate directly with clinical outcome, and the underlying reasons may be manifold: Although DHC creates an outlet for the herniating brain and reduces ICP, it does not prevent the formation of cytotoxic and vasogenic oedema [56]. The expansion of the brain comes at the cost of additional brain injury and progressive neurological deterioration caused by axonal shear stress, laceration of vessels and ischemia [35]. In addition, highly invasive approaches such as bifrontal craniectomy are frequently associated with a considerable rate of treatment-related complications and often necessitate a second surgery for the replacement of the bone flap [8, 56].

BC theoretically represents a physiological approach to the treatment of TBI-related prolonged ICP elevation by opening the cisterns to atmospheric pressure, relieving cisternal pressure, restoring glymphatic clearance, reversing the CSF-shift-oedema and reducing brain herniation [25, 58, 59]. Several previous case reports in the literature reported a beneficial effect of adjuvant BC on clinical outcomes following moderate to severe TBI [30, 34–36, 60, 61].

In our systematic review, we mainly identified studies comparing adjuvant BC (BC + DHC) versus standalone DHC, which allows to draw conclusions only about the additive effect of adjuvant BC (BC + DHC) in standalone DHC. Physiologically, standalone BC would constitute an adequate treatment of CSF-shift-oedema, however, proper comparative studies are lacking. As standalone DHC is an established last-resort treatment modality for refractory ICP, evaluating standalone BC in clinical studies would probably not be feasible without including DHC as the established treatment.

Concerning studies on adjuvant BC (BC + DHC) versus standalone DHC, there is evidence at least from randomized studies suggesting that in-hospital and long-term mortality are reduced with adjuvant BC (BC + DHC), however, neurological outcome was only non-significantly trending towards improvement for adjuvant BC (BC + DHC). While the mean GOS and the percentage of patients with GOS ≥ 5 at follow-up were consistently higher across all evaluated studies, likely due to the small sample size. Except for Kumar et al. [48], the majority of studies reported that patients with adjuvant BC (BC + DHC) had also lower mortality rates at final follow-up compared to patients with standalone DHC. Importantly, the study of Kumar et al. [48] suffers from several considerable limitations: the distribution of patients in the intervention groups was highly inequal, with only 9 patients in the adjuvant BC (BC + DHC) group and 31 patients in the standalone DHC group. Therefore, their study may be prone to random variation and overestimate the mortality rate in the adjuvant BC (BC + DHC) group, causing substantial heterogeneity in the meta-analysis. In contrast, the odds of mortality at follow-up were reduced by 60% when evaluating only the two relevant RCTs [50, 52], however, there is still insufficient evidence to draw valid conclusions.

Several additional factors may contribute to improved clinical outcome in patients with adjuvant BC (BC + DHC): Our meta-analysis demonstrated a notable reduction in the length of ICU stays in patients who underwent adjuvant BC (BC + DHC) compared to standalone DHC. In addition, there was a marked reduction in the odds of requiring osmotherapy in the adjuvant BC group (BC + DHC) – whose administration may itself potentially cause electrolyte abnormalities and renal failure [62].

It has been previously demonstrated that it is possible to drain a larger amount of CSF through the cisterns and thus achieve a more sustainable reduction of the ICP [27]. The rationale for an in-situ drain following BC is based on recent evidence suggesting that interstitial fluid communicates with cisternal CSF through the Robin-Virchow-spaces [58] – which may also explain why the placement of an external ventricular drainage has not shown many beneficial effects on clinical outcomes despite lowering the ICP [63, 64]. In our meta-analysis, the mean difference in the closing ICP and ICP in the ICU did not reach statistical significance as the two evaluated studies reported conflicting results.

From a technical point of view, basal durotomy followed by the release of CSF from the basal cisterns reduces cerebral edema and relaxes the brain relatively early during the procedure [65]. This allows a more gentle durotomy preventing kinking of cortical veins, lacerations of cerebral cortex on the bone edge and additional brain injury caused by cerebral swelling [66]. Importantly, the relaxation of the brain prevents herniation and enables the immediate reimplantation of the bone flap in some patients where it would otherwise have been impossible, thus avoiding the need for a second surgery for cranioplasty [29]. Our meta-analysis confirmed that patients undergoing adjuvant BC (BC + DHC) compared to standalone DHC had a significantly reduced mean brain outward herniation on postoperative CT-scans. In cases where a second procedure for cranioplasty was required, Giammattei et al. noted that the time to cranioplasty was shorter in patients who underwent adjuvant BC compared to DHC only [27].

While the reportedly positive effects of adjuvant BC (BC + DHC) on clinical outcome have encouraged neurosurgeons worldwide to advocate for its widespread adoption, there has been a resistance to implement BC as a complementary procedure in the treatment of TBI [26]. BC is a microneurosurgical procedure requiring training in vascular and skull base neurosurgery, appropriate instrumentation and the availability of a microscope [61]. Of note, TBI has the highest prevalence in developing countries where limited access to microneurosurgical equipment in trauma care centres may remain an obstacle for the widespread implementation of BC as a standard complementary procedure for TBI treatment [67]. In clinical practice, standalone DHC for TBI is frequently performed by neurosurgeons in training who may not always be able to safely perform adjuvant BC (BC + DHC) [68].

Due to considerable qualitative and clinical heterogeneity (including differences in inclusion criteria and clinical protocols) of the currently available evidence, it remains difficult to assess the effect of adjuvant BC (BC + DHC) on clinical outcome after TBI. As the majority of these studies originate from developing countries, it is also questionable to which extent those data would reflect the results of studies in Western healthcare systems. To determine the efficacy and safety of adjuvant BC (BC + DHC) in treatment of treatment of refractory hypertension following moderate to severe TBI, larger international RCTs are warranted.

### Limitations

Moderate to severe TBI summarizes a variety of pathologies with different prognoses which may itself cause a variance in outcomes. As a result, the heterogenous populations / inclusion criteria and varying indications may limit the comparability and generalizability of the studies. Due to the heterogeneity in patient cohorts and the low number of events reported outcomes, the evaluated studies are prone to random effects. The heterogeneity in study design limits the comparability of studies and decreased the number of eligible studies for meta-analysis to an average two to three, leading to a high statistical heterogeneity. Meta-analyses of just two or three studies therefore should be very cautiously interpreted. Including further databases and opting also for “gray literature” could expand the number of identified studies, potentially at the cost of study quality. Due to a considerable variance in the length of follow-up, our data may not allow for an accurate evaluation of final outcome of the patients. Similarly, different surgical protocols, e.g. adjuvant BC (BC + DHC) vs. standalone DHC, parcellate the available evidence and preclude drawing generalizable conclusions about the effects of these treatments with the currently limited published data. Overall, the abovementioned methodological limitations limit the ability to draw valid conclusions on the effect size of adjuvant BC (BC + DHC) on clinical outcome.

## Conclusion

Our systematic review and meta-analysis indicates that there is insufficient moderate- to high-quality data to demonstrate a potential beneficial effect of adjuvant BC (BC + DHC) on the clinical outcome following moderate to severe TBI. Despite some evidence for reduced mortality and length of stay, there is no effect on neurological outcome. However, these results need to be interpreted with caution as they carry a high risk of bias due to overall scarcity of published clinical data, technical variations, methodological differences, limited cohort sizes, and a considerable heterogeneity in study design and reported outcomes. Consequently, it remains difficult to draw valid and generalizable conclusions about the effect of adjuvant BC (BC + DHC) on clinical outcome.

## Electronic supplementary material

Below is the link to the electronic supplementary material.

Supplementary Material 1

Supplementary Material 2

Supplementary Material 3

## Data Availability

No datasets were generated or analysed during the current study.

## References

[1] Peeters W et al (2015) Epidemiology of traumatic brain injury in Europe, *Acta Neurochir. (Wien)*, vol. 157, fasc. 10, pp. 1683–1696, ott. 10.1007/s00701-015-2512-7 10.1007/s00701-015-2512-7 PMC4569652 26269030

[2] Murray GD et al (1999) mar., The European Brain Injury Consortium Survey of Head Injuries, *Acta Neurochir. (Wien)*, vol. 141, fasc. 3, pp. 223–236, 10.1007/s007010050292 10.1007/s007010050292 10214478

[3] Stocchetti N et al (2008) mar., Refractory intracranial hypertension and second-tier therapies in traumatic brain injury, *Intensive Care Med.*, vol. 34, fasc. 3, pp. 461–467, 10.1007/s00134-007-0948-9 10.1007/s00134-007-0948-9 18066523

[4] Ghajar J, injury Traumaticbrain (9233) *The Lancet*, vol. 356, fasc. pp. 923–929, set. 2000, 10.1016/S0140-6736(00)02689-1

[5] Waqas M et al (2016) mar., Predicting outcomes of decompressive craniectomy: use of Rotterdam Computed Tomography Classification and Marshall Classification, *Br. J. Neurosurg.*, vol. 30, fasc. 2, pp. 258–263, 10.3109/02688697.2016.1139047 10.3109/02688697.2016.1139047 26828246

[6] Brown AW et al (2019) apr., Predictive utility of an adapted Marshall head CT classification scheme after traumatic brain injury, *Brain Inj.*, vol. 33, fasc. 5, pp. 610–617, 10.1080/02699052.2019.1566970 10.1080/02699052.2019.1566970 PMC6436993 30663426

[7] Hutchinson PJ et al (2019) Consensus statement from the International Consensus Meeting on the Role of Decompressive Craniectomy in the Management of Traumatic Brain Injury: Consensus statement, *Acta Neurochir. (Wien)*, vol. 161, fasc. 7, pp. 1261–1274, lug. 10.1007/s00701-019-03936-y 10.1007/s00701-019-03936-y PMC6581926 31134383

[8] Hutchinson PJ et al (2016) Trial of Decompressive Craniectomy for Traumatic Intracranial Hypertension, *N. Engl. J. Med.*, vol. 375, fasc. 12, pp. 1119–1130, set. 10.1056/NEJMoa1605215 10.1056/NEJMoa1605215 27602507

[9] Jiang J-Y et al (2005) Efficacy of Standard Trauma Craniectomy for Refractory Intracranial Hypertension with Severe Traumatic Brain Injury: A Multicenter, Prospective, Randomized Controlled Study, *J. Neurotrauma*, vol. 22, fasc. 6, pp. 623–628, giu. 10.1089/neu.2005.22.623 10.1089/neu.2005.22.623 15941372

[10] Andrews PJD et al (2015) Hypothermia for Intracranial Hypertension after Traumatic Brain Injury, *N. Engl. J. Med.*, vol. 373, fasc. 25, pp. 2403–2412, dic. 10.1056/NEJMoa1507581 10.1056/NEJMoa1507581 26444221

[11] Roberts I (2004) Effect of intravenous corticosteroids on death within 14 days in 10 008 adults with clinically significant head injury (MRC CRASH trial): randomised placebo-controlled trial, *The Lancet*, vol. 364, fasc. 9442, pp. 1321–1328, ott. 10.1016/S0140-6736(04)17188-2 10.1016/S0140-6736(04)17188-2 15474134

[12] Mendelow AD et al (2015) Early Surgery versus Initial Conservative Treatment in Patients with Traumatic Intracerebral Hemorrhage (STITCH[Trauma]): The First Randomized Trial, *J. Neurotrauma*, vol. 32, fasc. 17, pp. 1312–1323, set. 10.1089/neu.2014.3644 10.1089/neu.2014.3644 PMC4545564 25738794

[13] Rush B, Rousseau J, Sekhon MS, Griesdale eDE (apr. 2016) Craniotomy Versus Craniectomy for Acute traumatic subdural hematoma in the United States: A National Retrospective Cohort Analysis. 88:25–31. World Neurosurg. 10.1016/j.wneu.2015.12.034 10.1016/j.wneu.2015.12.034 PMC4833577 26748175

[14] Yuan Q et al (2015) mar., Impact of intracranial pressure monitoring on mortality in patients with traumatic brain injury: a systematic review and meta-analysis, *J. Neurosurg.*, vol. 122, fasc. 3, pp. 574–587, 10.3171/2014.10.JNS1460 10.3171/2014.10.JNS1460 25479125

[15] Volovici V et al (2019) nov., Evolution of Evidence and Guideline Recommendations for the Medical Management of Severe Traumatic Brain Injury, *J. Neurotrauma*, vol. 36, fasc. 22, pp. 3183–3189, 10.1089/neu.2019.6474 10.1089/neu.2019.6474 31280663

[16] Volovici e V, Haitsma IK (2022) Cisternostomy in Traumatic Brain Injury: Time for the World to Listen—Cerebrospinal Fluid Release: Possibly the Missing Link in Traumatic Brain Injury, *World Neurosurg.*, vol. 162, pp. 3–5, giu. 10.1016/j.wneu.2022.02.121 10.1016/j.wneu.2022.02.121 35257952

[17] Bulat M, Klarica eM (2011) Recent insights into a new hydrodynamics of the cerebrospinal fluid, *Brain Res. Rev.*, vol. 65, fasc. 2, pp. 99–112, gen. 10.1016/j.brainresrev.2010.08.002 10.1016/j.brainresrev.2010.08.002 20817024

[18] Iliff JJ et al (2014) Impairment of Glymphatic Pathway Function Promotes Tau Pathology after Traumatic Brain Injury, *J. Neurosci.*, vol. 34, fasc. 49, pp. 16180–16193, dic. 10.1523/JNEUROSCI.3020-14.2014 10.1523/JNEUROSCI.3020-14.2014 PMC4252540 25471560

[19] Oresković D, Klarica eM The formation of cerebrospinal fluid: nearly a hundred years of interpretations and misinterpretations., pp. 241–262, 24 settembre 2010. 10.1016/j.brainresrev.2010.04.006 20435061

[20] Orešković D e, Klarica M (2014) A new look at cerebrospinal fluid movement, *Fluids Barriers CNS*, vol. 11, fasc. 1, p. 16, 10.1186/2045-8118-11-16 10.1186/2045-8118-11-16 PMC4118619 25089184

[21] Cherian I, Beltran M, Kasper E, Bhattarai B, Munokami S, Grasso eG (2016) Exploring the Virchow-Robin spaces function: A unified theory of brain diseases, *Surg. Neurol. Int.*, vol. 7, fasc. 27, p. 711, 10.4103/2152-7806.192486 10.4103/2152-7806.192486 PMC5093876 27857861

[22] Iliff JJ et al (2012) A Paravascular Pathway Facilitates CSF Flow Through the Brain Parenchyma and the Clearance of Interstitial Solutes, Including Amyloid β, *Sci. Transl. Med.*, vol. 4, fasc. 147, ago. 10.1126/scitranslmed.3003748 10.1126/scitranslmed.3003748 PMC3551275 22896675

[23] Cherian I, Beltran M, Landi A, Alafaci C, Torregrossa F, Grasso eG (2018) Introducing the concept of CSF-shift edema in traumatic brain injury, *J. Neurosci. Res.*, vol. 96, fasc. 4, pp. 744–752, apr. 10.1002/jnr.24145 10.1002/jnr.24145 28836291

[24] Cherian I, Grasso G, Bernardo A, Munakomi eS (2016) Anatomy and physiology of cisternostomy, *Chin. J. Traumatol.*, vol. 19, fasc. 1, pp. 7–10, feb. 10.1016/j.cjtee.2016.01.003 10.1016/j.cjtee.2016.01.003 PMC4897851 27033265

[25] Gaberel T et al (2014) Impaired Glymphatic Perfusion After Strokes Revealed by Contrast-Enhanced MRI: A New Target for Fibrinolysis?, *Stroke*, vol. 45, fasc. 10, pp. 3092–3096, ott. 10.1161/STROKEAHA.114.006617 10.1161/STROKEAHA.114.006617 25190438

[26] Cherian I, Yi G, Munakomi eS (2013) Cisternostomy: Replacing the age old decompressive hemicraniectomy?, *Asian J. Neurosurg.*, vol. 8, fasc. 03, pp. 132–138, set. 10.4103/1793-5482.121684 10.4103/1793-5482.121684 PMC3877499 24403955

[27] Giammattei L et al (2020) mar., Implementation of cisternostomy as adjuvant to decompressive craniectomy for the management of severe brain trauma, *Acta Neurochir. (Wien)*, vol. 162, fasc. 3, pp. 469–479, 10.1007/s00701-020-04222-y 10.1007/s00701-020-04222-y PMC7046565 32016585

[28] Parthiban JBC et al (2021) Basal Cisternostomy - A Microsurgical Cerebro Spinal Fluid Let Out Procedure and Treatment Option in the Management of Traumatic Brain Injury. Analysis of 40 Consecutive Head Injury Patients Operated with and without Bone Flap Replacement Following Cisternostomy in a Tertiary Care Centre in India, *Neurol. India*, vol. 69, fasc. 2, p. 328, 10.4103/0028-3886.314535 10.4103/0028-3886.314535 33904445

[29] Chandra VVR, Mowliswara Prasad BC, Banavath HN (2022) e K. Chandrasekhar Reddy, Cisternostomy versus Decompressive Craniectomy for the Management of Traumatic Brain Injury: A Randomized Controlled Trial, *World Neurosurg.*, vol. 162, pp. e58–e64, giu. 10.1016/j.wneu.2022.02.067 10.1016/j.wneu.2022.02.067 35192970

[30] Eraky AM, Treffy R, Hedayat eHS (2022) Cisternotomy and Liliequist’s membrane fenestration as a Surgical treatment for idiopathic intracranial hypertension (Pseudotumor Cerebri): a Case Report. Cureus Nov. 10.7759/cureus.31363 10.7759/cureus.31363 PMC9741810 36514638

[31] Peters DR et al (2023) nov., Cisternostomy for Severe Traumatic Brain Injury: Illustrative Case and Cadaveric Study of the Neurovascular Anatomy of the Basal Cisterns: 2-Dimensional Operative Video, *Oper. Neurosurg.*, vol. 25, fasc. 5, pp. e280–e281, 10.1227/ons.0000000000000835 10.1227/ons.0000000000000835 37527030

[32] Cherian I, Bernardo A, Grasso eG (2016) Cisternostomy for Traumatic Brain Injury: Pathophysiologic Mechanisms and Surgical Technical Notes, *World Neurosurg.*, vol. 89, pp. 51–57, mag. 10.1016/j.wneu.2016.01.072 10.1016/j.wneu.2016.01.072 26851743

[33] Garvayo M et al (2022) The positive impact of cisternostomy with cisternal drainage on delayed hydrocephalus after aneurysmal subarachnoid hemorrhage, *Acta Neurochir. (Wien)*, vol. 165, fasc. 1, pp. 187–195, dic. 10.1007/s00701-022-05445-x 10.1007/s00701-022-05445-x PMC9840569 36504078

[34] Encarnacion Ramirez MDJ, Barrientos Castillo RE, Vorobiev A, Kiselev N, Aquino AA, Efe eIE (2022) Basal cisternostomy for traumatic brain injury: A case report of unexpected good recovery, *Chin. J. Traumatol.*, vol. 25, fasc. 5, pp. 302–305, set. 10.1016/j.cjtee.2021.12.008 10.1016/j.cjtee.2021.12.008 PMC9458986 35033422

[35] El-Ghandour NMF (2023) Commentary: Cisternostomy for Severe Traumatic Brain Injury: Illustrative Case and Cadaveric Study of the Neurovascular Anatomy of the Basal Cisterns: 2-Dimensional Operative Video, *Oper. Neurosurg.*, vol. 25, fasc. 5, pp. e282–e283, nov. 10.1227/ons.0000000000000842 10.1227/ons.0000000000000842 37534904

[36] Eraky AM, Treffy R, Hedayat eHS (2023) Cisternostomy as a Surgical Treatment for Traumatic Brain Injury-related prolonged and delayed intracranial pressure elevation: a Case Report. Cureus Apr. 10.7759/cureus.37508 10.7759/cureus.37508 PMC10181949 37193467

[37] Moscote-Salazar LR, Narvaez-Rojas AR, Pacheco-Hernandez eA (2018) Cisternostomy: Surgical Alternative for Patients with Refractory Posttraumatic Intracranial Hypertension, *World Neurosurg.*, vol. 110, p. 507, feb. 10.1016/j.wneu.2017.10.160 10.1016/j.wneu.2017.10.160 29433168

[38] Mura J, Figueiredo E, Carmona P, Palma-Fellemberg Á (2013) e J. De Faria, The Anterior Ventriculo-Cisternostomy: The Pioneers’ Work Revisited, *J. Neurol. Surg. Part Cent. Eur. Neurosurg.*, vol. 74, fasc. 03, pp. 146–151, gen. 10.1055/s-0032-1330122 10.1055/s-0032-1330122 23315669

[39] *Covidence systematic review software VHI, Melbourne, Australia. Available at www.covidence.org.*

[40] Daudt HM, Van Mossel C, Scott eSJ (2013) Enhancing the scoping study methodology: a large, inter-professional team’s experience with Arksey and O’Malley’s framework, *BMC Med. Res. Methodol.*, vol. 13, fasc. 1, p. 48, dic. 10.1186/1471-2288-13-48 10.1186/1471-2288-13-48 PMC3614526 23522333

[41] Page MJ et al (2021) mar., The PRISMA 2020 statement: an updated guideline for reporting systematic reviews, *BMJ*, p. n71, 10.1136/bmj.n71 10.1136/bmj.n71 PMC8005924 33782057

[42] Wells GA, Wells G, Shea B et al The Newcastle-Ottawa Scale (NOS) for Assessing the Quality of Nonrandomised Studies in Meta-Analyses. 2014&#187

[43] Higgins JPT et al (2011) The Cochrane Collaboration’s tool for assessing risk of bias in randomised trials, *BMJ*, vol. 343, fasc. oct18 2, pp. d5928–d5928, ott. 10.1136/bmj.d5928 10.1136/bmj.d5928 PMC3196245 22008217

[44] Jpt H Cochrane handbook for systematic reviews of interventions. http://www.cochrane-handbook.orghttp://www.cochrane-handbook.org. 2008.

[45] Review Manager (RevMan) [Computer program]. Version 5.4. The Cochrane Collaboration

[46] Cherian I et al (2019) nov., Cisternostomy: A Timely Intervention in Moderate to Severe Traumatic Brain Injuries: Rationale, Indications, and Prospects, *World Neurosurg.*, vol. 131, pp. 385–390, 10.1016/j.wneu.2019.07.082 10.1016/j.wneu.2019.07.082 31658580

[47] Goyal N, Kumar eP (apr. 2021) Putting CSF-Shift Edema Hypothesis to test: comparing Cisternal and Parenchymal pressures after basal cisternostomy for Head Injury. World Neurosurg 148:e252–e263. 10.1016/j.wneu.2020.12.133 10.1016/j.wneu.2020.12.133 33412318

[48] Kumar P et al (2022) Basal Cisternostomy in Head Injury: More Questions than Answers, *Neurol. India*, vol. 70, fasc. 4, p. 1384, 10.4103/0028-3886.355117 10.4103/0028-3886.355117 36076632

[49] Encarnación M, Ramirez et al (2023) The Role of Cisternostomy in the Management of Severe Traumatic Brain Injury: A Triple-Center Study, *Surgeries*, vol. 4, fasc. 2, pp. 283–292, giu. 10.3390/surgeries4020029

[50] Singh A (2021) Surgical outcome of poor GCS patients of acute subdural hematoma with decompressive craniotomy alone v/s decompressive craniotomy with cisternostomy, *Clin. Med.*, vol. 08, fasc. 03

[51] Vemula RC, Prasad B, Banavath HN, Kale PKG, Krishna MM (2022) N, e S. Gokanapudi, Outcomes and Predictors of Outcome with Cisternostomy in the Management of Traumatic Brain Injury—A Prospective Observational Study at a Tertiary Centre, *Indian J. Neurotrauma*, vol. 19, fasc. 02, pp. 078–083, dic. 10.1055/s-0041-1739478

[52] Youssef O, Ali TM, Anbar K, El-Shahawy O, Enayet eA (2020) Value of Adding Cisternostomy to Decompressive Hemicraniectomy in the Management of Traumatic Acute Subdural Hematoma Patients, *Open Access Maced. J. Med. Sci.*, vol. 8, fasc. B, pp. 1014–1022, lug. 10.3889/oamjms.2020.4423

[53] Bruno A, Zahran A, Paletta N, Maali L, Nichols FT, Figueroa eR (mar. 2017) A standardized method to measure brain shifts with decompressive hemicraniectomy. J Neurosci Methods 280:11–15. 10.1016/j.jneumeth.2017.01.021 10.1016/j.jneumeth.2017.01.021 28163065

[54] Marmarou A (2007) A review of progress in understanding the pathophysiology and treatment of brain edema, *Neurosurg. Focus*, vol. 22, fasc. 5, pp. 1–10, mag. 10.3171/foc.2007.22.5.2 10.3171/foc.2007.22.5.2 17613227

[55] Khellaf A, Khan DZ, Helmy eA (2019) Recent advances in traumatic brain injury, *J. Neurol.*, vol. 266, fasc. 11, pp. 2878–2889, nov. 10.1007/s00415-019-09541-4 10.1007/s00415-019-09541-4 PMC6803592 31563989

[56] Capizzi A, Woo J, Verduzco-Gutierrez eM, Injury TraumaticB (2020) *Med. Clin. North Am.*, vol. 104, fasc. 2, pp. 213–238, mar. 10.1016/j.mcna.2019.11.001 10.1016/j.mcna.2019.11.001 32035565

[57] Valle D, Villarreal X, Lunny eC (2022) Surgical Management of Neurotrauma: When to Intervene., pp. 41–55, dicembre

[58] Yang L et al (2013) Evaluating glymphatic pathway function utilizing clinically relevant intrathecal infusion of CSF tracer, *J. Transl. Med.*, vol. 11, fasc. 1, p. 107, dic. 10.1186/1479-5876-11-107 10.1186/1479-5876-11-107 PMC3665671 23635358

[59] Ringstad G, Vatnehol SAS, Eide ePK (2017) Glymphatic MRI in idiopathic normal pressure hydrocephalus, *Brain*, vol. 140, fasc. 10, pp. 2691–2705, ott. 10.1093/brain/awx191 10.1093/brain/awx191 PMC5841149 28969373

[60] Masoudi M, Rezaee E, Hakiminejad H, Tavakoli M, Sadeghpoor eT Cisternostomy for Management of Intracranial Hypertension in Severe Traumatic Brain Injury; Case Report and Literature Review., pp. 161–4, luglio 2016 PMC4989043 27540551

[61] Villanueva P et al (mar. 2023) Microneurosurgical anatomy of the basal cisterns: a brief review for cisternostomy. Surg Neurol Int 14:97. 10.25259/SNI_1095_2022 10.25259/SNI_1095_2022 PMC10070334 37025519

[62] Rangel-Castillo L, Gopinath S, Robertson eCS (2008) Management of Intracranial Hypertension, *Neurol. Clin.*, vol. 26, fasc. 2, pp. 521–541, mag. 10.1016/j.ncl.2008.02.003 10.1016/j.ncl.2008.02.003 PMC2452989 18514825

[63] Volovici V et al (2019) apr., Ventricular Drainage Catheters versus Intracranial Parenchymal Catheters for Intracranial Pressure Monitoring-Based Management of Traumatic Brain Injury: A Systematic Review and Meta-Analysis, *J. Neurotrauma*, vol. 36, fasc. 7, pp. 988–995, 10.1089/neu.2018.6086 10.1089/neu.2018.6086 30251919

[64] Timofeev I et al (2008) Ventriculostomy for control of raised ICP in acute traumatic brain injury, in *Acta Neurochirurgica Supplements*, vol. 102, H.-J. Steiger, A c. di, in Acta Neurochirurgica Supplementum, vol. 102., Vienna: Springer Vienna, pp. 99–104. 10.1007/978-3-211-85578-2_20 10.1007/978-3-211-85578-2_20 19388297

[65] Alves OL, Bullock eR (2003) ?Basal durotomy? to prevent massive intra-operative traumatic brain swelling, *Acta Neurochir. (Wien)*, vol. 145, fasc. 7, pp. 583–586, gen. 10.1007/s00701-003-0055-9 10.1007/s00701-003-0055-9 12910402

[66] Jiang Y-Z, Lan Q, Wang Q-H, Song D-L, Lu H (2014) e W.-J. Wu, Gradual and Controlled Decompression for Brain Swelling Due to Severe Head Injury, *Cell Biochem. Biophys.*, vol. 69, fasc. 3, pp. 461–466, lug. 10.1007/s12013-014-9818-6 10.1007/s12013-014-9818-6 24442991

[67] Kanmounye US (2021) The Rise of Inflow Cisternostomy in Resource-Limited Settings: Rationale, Limitations, and Future Challenges, *Emerg. Med. Int.*, vol. pp. 1–4, gen. 2021, 10.1155/2021/6630050 10.1155/2021/6630050 PMC7810553 33505727

[68] Di Cristofori A, Gerosa A, Panzarasa eG (2018) Is Neurosurgery Ready for Cisternostomy in Traumatic Brain Injuries?, *World Neurosurg.*, vol. 111, p. 427, mar. 10.1016/j.wneu.2017.11.139 10.1016/j.wneu.2017.11.139 29499598

