# Supplementary material for: Basal cisternostomy as an adjunct to decompressive hemicraniectomy in moderate to severe traumatic brain injury: a systematic review and meta-analysis
Source: Neurosurg Rev. 2024 Oct 2;47(1):717. doi: 10.1007/s10143-024-02954-4 (PMC11445355; doi:10.1007/s10143-024-02954-4)
Supplement: Supplementary file 1 — Supplementary Material 1 [file 10143_2024_2954_MOESM1_ESM.docx]

**Supplementary Content 1**

**Supplementary Table 1:** Study characteristics.

| **Author** | **No. Pts** | **Study type** | **Methods** | **Partcipants** | **Interventions** | **Outcomes** |
| --- | --- | --- | --- | --- | --- | --- |
| **Adjuvant BC (BC + DHC) vs. standalone DHC** | | | | | | |
| **Kumar et al., 2022** [48] | 40 | Prospective;  Quasi-experimental (according to which surgeon was on call);  Single-center | **Methods of randomization:** surgeon on-call  **Blinding of outcome assessment:** no  **Analysis**: treatment received  **Excluded participants**: no  **Cross-over cases**: 0  **Losses to follow-up**: 0  **Definition of outcomes**: stated | **Location:**  Department of Neurosurgery, All India Institute of Medical Sciences, Veerbhandra Road, Rishikesh, Uttarakhand, India  **Groups**:  BC+DHC, DHC  **Age**:  *BC*+*DHC*: 45.7±14.0  *DHC*: 39.2±16.0  **Inclusion criteria.**  Thickness≥10 mm, Midline shift≥5 mm | *BC*+*DHC*: 9 patients  *DHC*: 31 patients | ICU stay  Length of hospital stay  GCS at discharge  GOS-E≥5 at 30 days follow-up  Mortality at 30 days follow-up |
| **Youssef et al., 2020** [52] | 40 | Prospective; RCT; single-center | **Methods of randomization**: successive numbers  **Blinding of outcome assessment**: no  **Analysis**: intention to treat  **Excluded participant**s: 0  **Cross-over cases**: 0  **Losses to follow-up**: 0  **Definition of outcomes**: stated | **Location**:  Department of Neurosurgery, Faculty of Medicine, Cairo University, Cairo, Egypt  **Groups**:  BC+DHC, DHC  **Age**:  *BC+DHC*: 39.7±15.5  *DHC*: 41.2±14.3  **Inclusion criteria**. A  SDH, Thickness≥10 mm, Midline shift≥5 mm, GCS<10 | *BC*+*DHC*: 20 patients  *DHC*: 20 patients | ICU stay in days  Brain outward herniation  Patients requiring osmotherapy  Mean GOS at 4 weeks follow-up  Mortality at discharge  Mortality at 6 months follow-up |
| **Singh et al., 2021** [50] | 54 | Prospective;  RCT;  single-center | **Methods of randomization:**  not known  **Blinding of outcome assessment:** not known  **Analysis:** intention to treat  **Excluded participants**: 0  **Cross-over cases:** 0  **Losses to follow-up**: 0  **Definition of outcomes**: stated | **Location**:  Maharashi Mahakandeshwar Institute of  Medical Science and Research,Mullana,Haryana, India  **Groups**:  BC+DHC, DHC  **Age:**  *BC+DHC:*  50.33±17  *DHC:*  42.25±11  **Inclusion criteria:**  aSDH, Severe TBI (GCS≤7) | *BC*+*DHC*: 27 patients  *DHC*: 27 patients | Brain outward herniation  Patients requiring osmotherapy  Closing ICP  ICP in the ICU  ICU stay in days  GOS≥5 at follow-up  Mortality at discharge  Mortality at 6 months follow-up |
| **Giammattei et al., 2020** [27] | 40 | Retrospective; single-center | **Methods of randomization**: not applicable  **Blinding of outcome assessment**: no  **Analysis**: treatment received  **Excluded participants:** 0  **Cross-over cases**: 0  **Losses to follow-up**: 2  **Definition of outcomes**: stated | **Location**:  Department of Clinical Neurosciences, Service of Neurosurgery,  Lausanne University Hospital (CHUV), Lausanne, Switzerland  **Groups**:  *BC*+*DHC*, *DHC*  **Age**:  *BC+DHC*: 49.9±19  *DHC*: 48.4±20.4  **Inclusion criteria:** BC+DHC:Severe TBI (GCS≤8), aSDH, aEDH, aICH ≥10 mm  DHC: Severe TBI (GCS≤8), aSDH≥10 mm | *BC*+*DHC*: 18  *DHC*: 22 | Duration of Closing ICP  ICP in the ICU  ICU stay in days  Brain outward herniation  Patients requiring osmotherapy  GOS≥5 at 6 months follow-up  Mortality at discharge  Mortality at 6 months follow-up |
| **Adjuvant BC (BC+DHC) vs. standalone BC** | | | | | | |
| **Parthiban et al., 2021** [28] | 40 | Retrospective; single-center, single surgeon | **Methods of randomization**: not applicable  **Blinding of outcome assessment**: no  **Analysis**: treatment received  **Excluded participants**: 0  **Cross-over cases**: 0  **Losses to follow-up**: 0  **Definition of outcomes**: stated | **Location**: Department of Neurosurgery, Kovai Medical Center and Hospital, Coimbatore, Tamil Nadu, India  **Groups**:  *BC*+*DHC*, *BC*  **Age**:  44.3 (range 17-70)  **Inclusion criteria:**  Not specified | *BC*+*DHC*: 13  *BC*: 27 | GOS≥4 at 6-months follow-up  Complications  Length of hospital stay |
| **Encarnación Ramirez et al., 2023** [49] | 30 | Prospective; multicenter | **Methods of randomization**: not applicable  **Blinding of outcome assessment**: no  **Analysis**: treatment received  **Excluded participants**: 0  **Cross-over cases**: 0  **Losses to follow-up**: 0  **Definition of outcomes**: stated | **Location**:  RUDN University (Moscow, Russia), General Hospital (Durango, Mexico), and Hospital Regional Universitario José María Cabral y Baez (Santiago, Dominican Republic)  **Groups**:  *BC*+*DHC*, *BC*  **Age**:  Not specified  **Inclusion criteria**:  Age 18-70 years, Severe TBI (GCS≤8), DSH > 20mm or Midline shift > 5mm on CT | *BC*+*DHC*: 24  *BC*: 6 | GOS≥5 at 6-months follow-up  Mortality at dicharge |
| **Adjuvant BC (BC+DHC) vs. standalone BC vs. standalone DHC** | | | | | | |
| **Cherian et al., 2019** [46] | 1032 | Retrospective; single-center | **Methods of randomization**: not applicable  **Blinding of outcome assessment**: no  **Analysis**: treatment received  **Excluded participants**: 0  **Cross-over cases**: 0  **Losses to follow-up**: 0  **Definition of outcomes**: stated | **Location**:  Department of Neurosurgery, Institute of Neurosciences, Nobel Medical College and Teaching Hospital, Biratnagar, Nepal;  **Groups**:  *BC*+*DHC*, *BC*, *DHC*  **Age:**  *BC*: 44.48± 12.48  *DHC*: 42.84±13.90  **Inclusion criteria**:  MPC score 3-7 (scale 0-10), GCS motor score M4, Anisocoria (unilaterally dilatative and reactive pupil), Obliteration of suprasellar cisterns/cerebellopontine angle cistern widening, Oculomotor nerve palsy, tSAH with brain swelling, aSDH, EDH with brain swelling or mass effect | *BC*+*DHC*: 272 patients  *BC*: 476 patients  *DHC*: 284 patients | GOS-E≥5 at 6 weeks follow-up  Mortality at discharge  Length of hospital stay |
| **Adjuvant BC (BC + DHC) standalone** | | | | | | |
| **Goyal and Kumar, 2021** [47] | 9 | Prospective; observational; single-center | **Methods of randomization**: not applicable  **Blinding of outcome assessment**: no  **Analysis**: treatment received  **Excluded participants**: 0  **Cross-over cases**: 0  **Losses to follow-up**: 0  **Definition of outcomes**: stated | **Location**:  Department of Neurosurgery, All India Institute of Medical Sciences, Rishikesh, India  **Groups**:  *BC*+*DHC*  A**ge range**:  45.7±14.04 (range 25-72)  **Inclusion criteria:**  Not specified | *BC*+*DHC*: 9 patients | Closing ICP  ICP in the ICU  Complications |

aSDH acute subdural hematoma; DHC, standalone decompressive hemicraniectomy; BC+DHC, adjuvant basal cisternostomy + decompressive hemicraniectomy; EDH, epidural hematoma; ICH, intracerebral hematoma, RCT, randomized controlled trial; SD, standard deviation; TBI traumatic brain injury; tSAH, traumatic subarachnoid hemorrhage
