# Supplementary material for: Basal cisternostomy as an adjunct to decompressive hemicraniectomy in moderate to severe traumatic brain injury: a systematic review and meta-analysis
Source: Neurosurg Rev. 2024 Oct 2;47(1):717. doi: 10.1007/s10143-024-02954-4 (PMC11445355; doi:10.1007/s10143-024-02954-4)
Supplement: Supplementary file 2 — Supplementary Material 2 [file 10143_2024_2954_MOESM2_ESM.docx]

**Supplementary Content 2 – Secondary Results 1**

**Secondary Comparisons:**

**Adjuvant BC (BC + DHC) vs standalone BC**

In a retrospective single-center study by Parthiban et al. [28] comparing standalone BC and adjuvant BC (BC + DHC), patients with standalone BC had higher rates of GOS ≥4 (85% (23/27) vs. 77% (10/13)) compared to adjuvant BC (BC + DHC) as well as lower mortality rates (4% (1/27) vs. 8% (1/13)) at six months follow-up. In their prospective, triple-center study, Encarnación Ramirez et al. [49], patients with standalone BC had a 100% (5/5) rate of GOS-E ≥5 compared to 91.7% (19/21) rate in patients with adjuvant BC (BC + DHC).

**Adjuvant BC (BC + DHC) versus standalone BC versus standalone DHC**

In their retrospective single-center study comprising a total of 1032 patients, Cherian et al. [46] reported higher mean GOS (3.9 vs. 3.7 vs. 2.8) at six weeks follow-up, lower mortality rates (16% (74/476) vs. 26% (72/272) vs. 35% (99/284)) and shorter length of stay in the ICU (2 days vs. 3 days vs. 6 days) in patients with adjuvant BC (BC + DHC) and standalone BC compared with standalone DHC.

**Adjuvant BC (BC + DHC)**

At seventy-two hours postoperatively, nine patients undergoing adjuvant BC (BC + DHC) in a study by Goyal and Kumar [47] had a mean (SD) GCS of 5.67±3.78 and a mortality rate of 30% (3/9). The mean (SD) ICP decreased from 25.70±10.48 mmHg after the first burr hole to 11.30±5.95 mmHg at skin closure. In the first 72 hours after surgery, the mean (SD) cisternal and parenchymal pressures were 32±19.95 mmHg and 20.17±21.58 mmHg, respectively.
