# Supplementary material for: Basal cisternostomy as an adjunct to decompressive hemicraniectomy in moderate to severe traumatic brain injury: a systematic review and meta-analysis
Source: Neurosurg Rev. 2024 Oct 2;47(1):717. doi: 10.1007/s10143-024-02954-4 (PMC11445355; doi:10.1007/s10143-024-02954-4)
Supplement: Supplementary file 3 — Supplementary Material 3 [file 10143_2024_2954_MOESM3_ESM.docx]

**Supplementary Content 3**


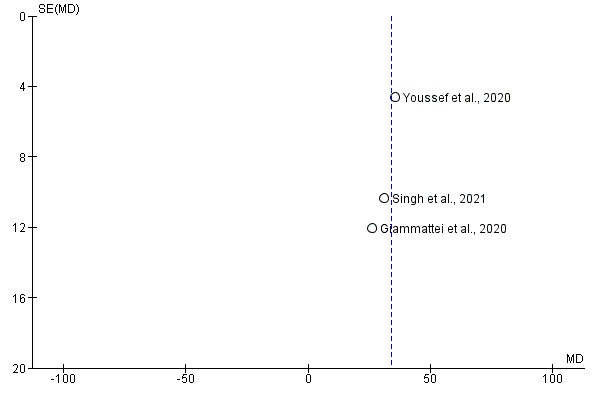
**Supplementary Figure 1***: Funnel plot Duration of Surgery in Adjuvant BC (BC + DHC) versus standalone DHC (all studies).*


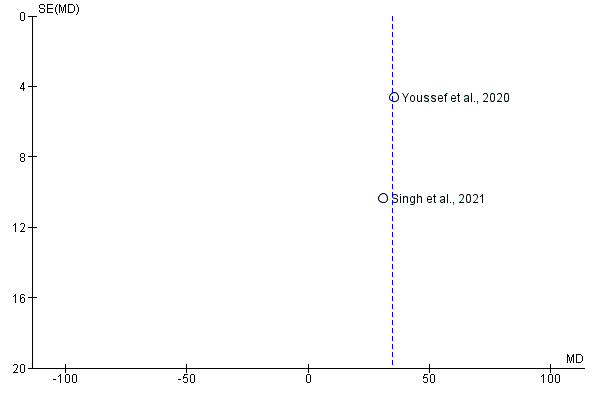
**Supplementary Figure 2**: *Funnel plot Duration of Surgery in Adjuvant BC (BC + DHC) versus Standalone DHC (RCT only)*.


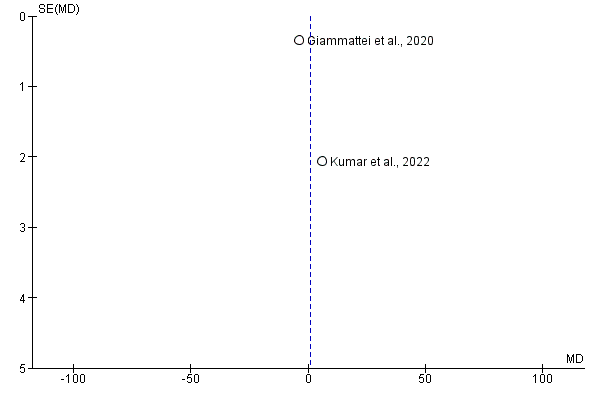
**Supplementary Figure 3**: *Funnel plot Closing ICP in Adjuvant BC (BC + DHC) versus Standalone DHC*.


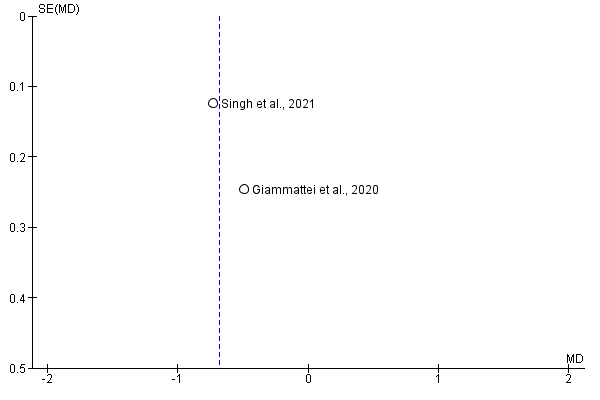
**Supplementary Figure 4**: *Funnel plot Brain Outward Herniation in Adjuvant BC (BC + DHC) versus Standalone DHC*.

**Supplementary Figure 5:** *Funnel plot Length of Stay in the ICU in Adjuvant BC (BC + DHC) versus Standalone DHC (All Studies).*


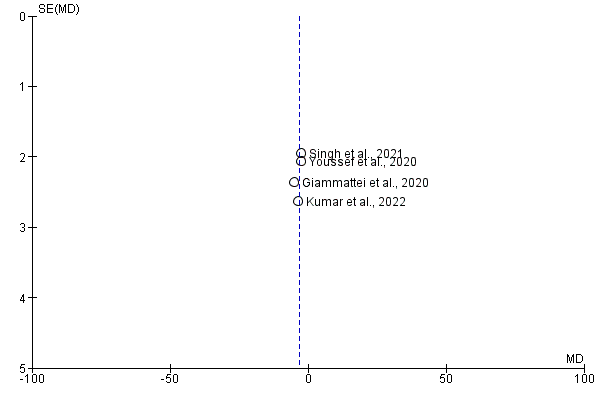


*
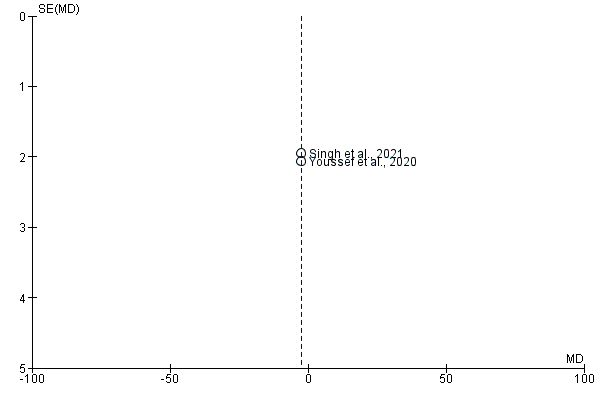
***Supplementary Figure 5**: *Funnel plot Length of Stay in the ICU in Adjuvant BC (BC + DHC) versus Standalone DHC (RCT only).*

*
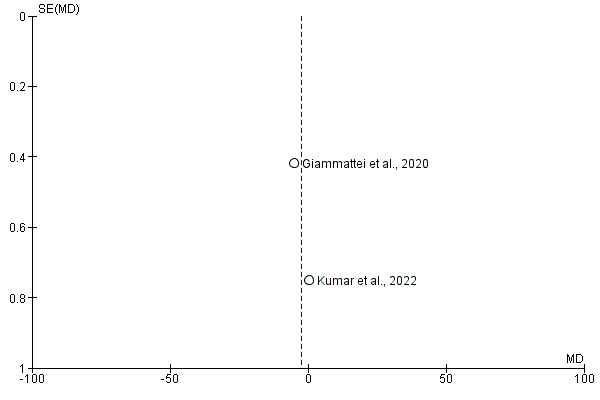
***Supplementary Figure 6**: *Funnel plot ICP in the ICU in Adjuvant BC (BC + DHC) versus Standalone DHC.*

*
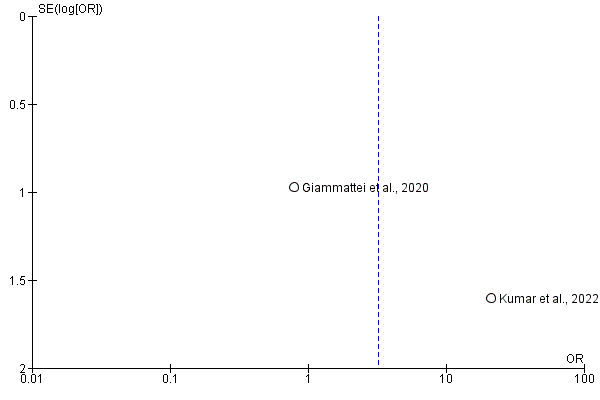
***Supplementary Figure 7**: *Funnel plot Complications in Adjuvant BC (BC + DHC) versus Standalone DHC.*


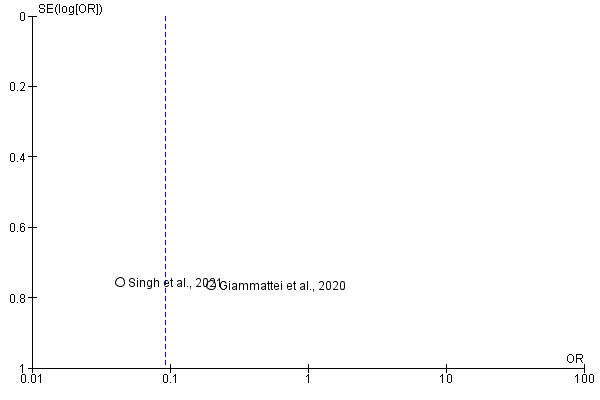
**Supplementary Figure 8**: *Funnel plot N. of patients requiring osmotherapy in Adjuvant BC (BC + DHC) versus Standalone DHC.*

**
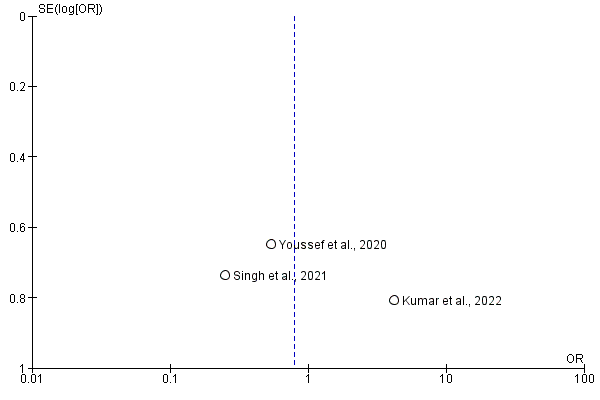
Supplementary Figure 9**: *Funnel plot N. of Mortality at follow up (All Studies) in Adjuvant BC (BC + DHC) versus Standalone DHC*

*
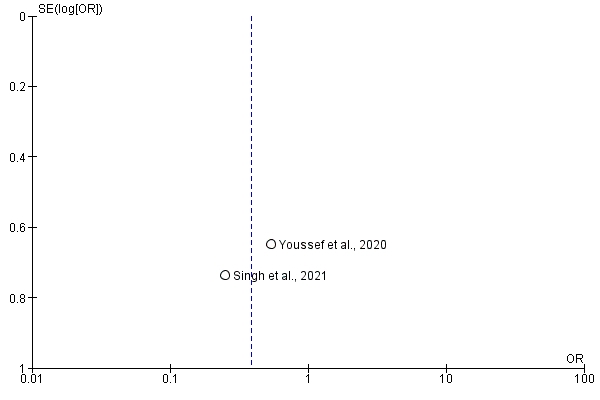
***Supplementary Figure 10**: *Funnel plot N. of Mortality at follow up (RCT Only) in Adjuvant BC (BC + DHC) versus Standalone DHC.*

**Supplementary Figure 11**: *Funnel plot GOS ≥ 5 at follow up in Adjuvant BC (BC + DHC) versus Standalone DHC.*

*
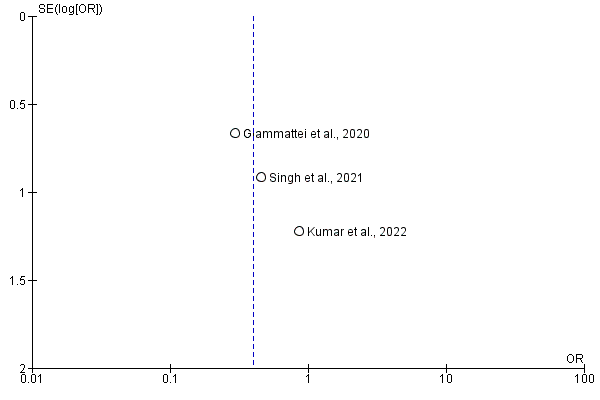
*

*
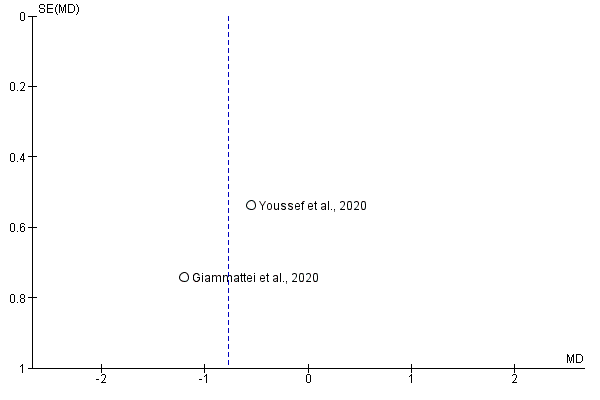
***Supplementary Figure 12**: *Mean GOS at follow up in Adjuvant BC (BC + DHC) versus Standalone DHC.*
